# Enzymatic machinery of wood-inhabiting fungi that degrade temperate tree species

**DOI:** 10.1093/ismejo/wrae050

**Published:** 2024-03-22

**Authors:** Lydia Kipping, Nico Jehmlich, Julia Moll, Matthias Noll, Martin M Gossner, Tim Van Den Bossche, Pascal Edelmann, Werner Borken, Martin Hofrichter, Harald Kellner

**Affiliations:** Department of Molecular Toxicology, Helmholtz-Centre for Environmental Research—UFZ GmbH, 04318 Leipzig, Germany; Institute for Bioanalysis, University of Applied Sciences Coburg, 96450 Coburg, Germany; Department of Molecular Toxicology, Helmholtz-Centre for Environmental Research—UFZ GmbH, 04318 Leipzig, Germany; Department of Soil Ecology, Helmholtz Centre for Environmental Research—UFZ GmbH, 06120 Halle (Saale), Germany; Institute for Bioanalysis, University of Applied Sciences Coburg, 96450 Coburg, Germany; Department of Soil Ecology, University of Bayreuth, 95448 Bayreuth, Germany; Forest Entomology, Swiss Federal Research Institute WSL, 8903 Birmensdorf, Switzerland; Department of Environmental Systems Science, Institute of Terrestrial Ecosystems, ETH Zürich, 8092 Zürich, Switzerland; VIB—UGent Center for Medical Biotechnology, VIB, 9052 Ghent, Belgium; Department of Biomolecular Medicine, Faculty of Medicine and Health Sciences, Ghent University, 9052 Ghent, Belgium; Department of Ecology and Ecosystem Management, Center of School of Life and Food Sciences Weihenstephan, TU München, 85354 Freising, Germany; Department of Soil Ecology, University of Bayreuth, 95448 Bayreuth, Germany; Department of Bio- and Environmental Sciences, International Institute Zittau, TU Dresden, 02763 Zittau, Germany; Department of Bio- and Environmental Sciences, International Institute Zittau, TU Dresden, 02763 Zittau, Germany

**Keywords:** metaproteomics, lignocellulolytic enzymes, deadwood decomposition, Ascomycota, Basidiomycota, angiosperms, gymnosperms, functional redundancy, simultaneous, decay, species richness

## Abstract

Deadwood provides habitat for fungi and serves diverse ecological functions in forests. We already have profound knowledge of fungal assembly processes, physiological and enzymatic activities, and resulting physico-chemical changes during deadwood decay. However, *in situ* detection and identification methods, fungal origins, and a mechanistic understanding of the main lignocellulolytic enzymes are lacking. This study used metaproteomics to detect the main extracellular lignocellulolytic enzymes in 12 tree species in a temperate forest that have decomposed for 8 ½ years. Mainly white-rot (and few brown-rot) Basidiomycota were identified as the main wood decomposers, with *Armillaria* as the dominant genus; additionally, several soft-rot xylariaceous Ascomycota were identified. The key enzymes involved in lignocellulolysis included manganese peroxidase, peroxide-producing alcohol oxidases, laccase, diverse glycoside hydrolases (cellulase, glucosidase, xylanase), esterases, and lytic polysaccharide monooxygenases. The fungal community and enzyme composition differed among the 12 tree species. Ascomycota species were more prevalent in angiosperm logs than in gymnosperm logs. Regarding lignocellulolysis as a function, the extracellular enzyme toolbox acted simultaneously and was interrelated (e.g. peroxidases and peroxide-producing enzymes were strongly correlated), highly functionally redundant, and present in all logs. In summary, our *in situ* study provides comprehensive and detailed insight into the enzymatic machinery of wood-inhabiting fungi in temperate tree species. These findings will allow us to relate changes in environmental factors to lignocellulolysis as an ecosystem function in the future.

## Graphical Abstract

**Graphical Abstract:**
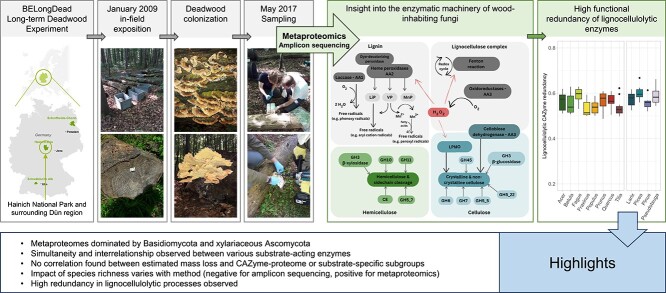

## Introduction

Microbial communities exhibit overwhelming diversity; how this diversity relates to ecosystem functioning has been intensely researched, but many aspects remain unknown. Metaproteomics is a powerful tool for revealing the hidden aspects of microbial communities and functions in environmental samples. This sophisticated method can link species diversity to activities and ultimately to resulting ecosystem processes [1]. Metaproteomics has been primarily applied to describe the functionality of microbes, mainly prokaryotic communities, in studies using pure or enriched cultures and less so in *in situ* studies [2, 3], with only a few examples for fungi [4].

Deadwood is a complex substrate and a crucial component of forests, sequestering 73 ± 6 Pg or ~8% of the global terrestrial carbon stock [5]. It provides habitat and nutrients for numerous specialized organisms, including deadwood-colonizing fungi, bacteria, archaea, arthropods, and nematodes. Intensive surveys and amplicon sequencing have steadily increased our understanding of microbial and invertebrate diversity in deadwood [6-9]. It has become evident that varying wood properties among tree species and exogenous parameters such as climate, soil traits, and forest structure affect colonization and decay dynamics in temperate forests [6, 10-12].

Deadwood, which has a low nutrient content and a recalcitrant lignin barrier that protects polysaccharides from decomposition, is difficult to exploit as a resource for most microorganisms. Filamentous fungi have well-adapted decomposition strategies, making them the dominant decomposers for this substrate. Depending on the target polymers and the color of the wood residues, these fungi are generally divided into four ecological groups: (i) white-rot fungi (WRF) (Basidiomycota), which attack lignin, metabolize hemicelluloses and some cellulose but leave behind whitish fibers; (ii) brown-rot fungi (BRF) (Basidiomycota), which attack and metabolize cellulose, including crystalline moieties and hemicelluloses, and leave behind mostly brownish lignin cubicles; (iii) soft-rot fungi (SRF) (Ascomycota), which attack cellulose, and to a lesser extent lignin, and metabolize hemicelluloses, leaving behind a brownish grey, spongy wood-like structure; and (iv) wood-associated fungi, which are mostly commensal and belong to Ascomycota, utilize low-molecular-mass wood ingredients and the products of wood decomposers [13, 14]. Each ecological group of fungi has specific underlying mechanisms, including the use of specific extracellular enzymes and radical-based oxidation mechanisms, which are also evident in differences in their genomes [15-17]. In laboratory studies, WRF were found to use a variety of peroxidases, peroxide (H_2_O_2_)-producing enzymes, laccases, lytic-polysaccharide monooxygenases (LPMOs), glycoside hydrolases (GHs), and esterases to simultaneously break down lignin, hemicelluloses, and cellulose [13, 18, 19]. In contrast, BRF mainly use hydroxyl radicals (^●^OH) released by Fenton reactions and a few hydrolases to attack cellulose and hemicelluloses [20, 21]. Furthermore, xylariaceous SRF contribute to cellulose and hemicellulose decomposition via diverse GHs, and they also partially degrade some lignin via the use of feruloyl esterase and laccase [22]. Enzymes that act on polymeric cell wall polysaccharides and lignin are classified in the CAZy database [23]. Various specialized wood-inhabiting fungi have been observed, isolated, and genome-sequenced [15-17], or their proteomes have been analyzed [24]. These findings provide the necessary basis for metaproteomic studies of the fungal enzyme repertoire and associated activities in environmental samples.

The decomposition process is divided into different stages marked by changes in fungal communities, ecological guilds, and wood quality [25-27]. Molecular taxonomy-based studies have revealed strong community differences between host tree species, indicating that specialized organisms contribute to the decomposition process [28, 29]. The relatively high number of wood colonizers and their changes during the decay process, along with the specificity of fungal taxa, raise questions about how the fungal community composition is related to the decomposition process and whether widespread functional redundancy occurs. Functional redundancy is the ability of different microbial taxa to carry out equal or similar functions [30, 31]. This theory assumes that shifts in community composition do not affect ecosystem processes as different members of the same functional group would replace each other. Knowledge of functional redundancy during the decomposition process of wood, especially regarding fungal contributions and the lignocellulolytic digestion enzymes deployed *in situ*, is scarce and needs clarification [32, 33]. Forest ecosystems are under pressure from anthropogenic impacts, and understanding the underlying molecular mechanisms, particularly the enzymatic mechanisms in their natural environment and their ecosystem relevance to higher levels, such as carbon sequestration, is important.

The main objective of this study was to determine the complex relationship between fungi and their extracellular lignocellulolytic enzyme machinery during deadwood decomposition. To achieve this goal, we applied an *in situ* metaproteomic approach to the deadwood of twelve common tree species after ~8 ½ years of decay in a temperate forest. This experiment is part of a long-term deadwood experiment with a focus on biodiversity, management, and ecosystem processes [10]. We aimed to identify the key fungal drivers involved in deadwood decomposition and to characterize the types of lignocellulolytic enzymes they employ across 12 different tree species. We investigated the impact of mass loss in individual logs on the fungal metaproteome and explored the correlation between fungal richness and their metaproteome. Because of the presence of numerous active fungal species after ~8 ½ years of wood decomposition, we also investigated if functional redundancy within the lignocellulolytic enzymatic machinery occurs *in situ*.

## Methods

### Study area and deadwood sampling

This study was conducted within the Biodiversity-Exploratories Long-term Deadwood (BELongDead) Experiment, which is an interdisciplinary effort investigating the diversity and function of deadwood-inhabiting organisms during the entire decay process [10]. BELongDead is part of the DFG-funded research platform Biodiversity-Exploratories in three German regions with standardized study plots [34], where logs of 12–13 tree species (from one donor location) with a diameter of 30–40 cm and length of 4 m were placed in 100 × 100 m forest plots at the beginning of 2009 (official start: 1. January 2009). For this study, wood samples were collected from 12 plots in one region (Hainich National Park and surrounding Dün; 51.097324N 10.458212E) in central Germany in May 2017 after ~8 ½ years of exposure. The region covers an area of 1300 km^2^, ranges between 285 and 550 m above sea level, and is one of the largest continuous deciduous forest areas in central Germany. Because of its status as a strictly protected area, most of the forest stands are not used for forestry. The main tree species, *Fagus sylvatica*, covers 83% of the forest area, whereas the remaining 17% is coniferous forest dominated by *Picea abies* [34].

Wood chips of 12 temperate tree species (eight angiosperms and four gymnosperms) per 12 plots were collected without bark using a power drill with a 2-cm auger 25 cm in length (Supplementary Table S1). Between each sampling, the auger was flame sterilized. Because of losses during laboratory handling and subsequent processing, a total of 127 wood samples were further subjected to metaproteomic and amplicon sequencing of the microbial community.

### Estimation of mass loss

Mass loss was estimated for individual logs based on decay functions published previously [35] as part of the entire BELongDead Experiment encompassing 1066 logs. By recording the density, mass, and volume loss of the exposed logs, the mass loss of all individual logs was calculated as the relative difference (%) between January 2009 and October/November 2018 (9.8 years). By determining the deadwood densities of the logs in 2012, 2015, and 2018, the mathematical models were refined to calculate the mass loss over time. The sigmoidal model best described the mass loss of *Fraxinus*, *Pinus,* and *Quercus*, whereas the linear model was best for all the other tree species. Based on these models, we estimated the relative mass loss separately for our 127 samples after ~8 ½ years of exposure.

### Amplicon sequencing for proteomics database selection

An amplicon sequencing approach using standard fungal primers [11, 36] was performed to (i) identify the most abundant wood-decomposing fungi as the basis for the metaproteomic database and (ii) compare both meta-approaches. Briefly, genomic DNA was isolated from ~100 mg of each milled wood sample (i.e. a fine powder) using a Quick-DNA Fecal/Soil Microbe Kit (Zymo Research, Irvine, CA, USA). The fungal ITS2 region was amplified using the primers fITS7/ITS4 and sequenced using MiSeq (Illumina, Inc., San Diego, CA, USA). Amplified sequence variants (ASVs) were generated using DADA2 [37] implemented in the “dadasnake” pipeline [38] as previously described [39], but the maximum number of “expected errors” was set to 2, and the reads were truncated at the first nucleotide with a quality score of 10. To consider potential intraspecific sequence variation, fungal ASVs were clustered into operational taxonomic units (OTUs) at 97% sequence similarity using VSEARCH [40]. Taxonomy was assigned using the Bayesian classifier implemented in “mothur” [41] against the UNITE database (version 9.0) [42], and fungal ecology was assigned using “FungalTrait” [43].

### Protein extraction and sample preparation

The collected wood chips were handled according to a previously described procedure [44] (exhaustively described in the Supplementary Methods). Briefly, the wood chips were fine milled and extracted using a cell lysis step that included thaw–freeze cycles and ultrasonication. Phenolic extraction was then performed, followed by subsequent precipitation, centrifugation, and pellet-washing steps. The pellets were dissolved in SDS buffer, heated, and separated via SDS–PAGE. The protein bands were cut from the SDS–PAGE gel, destained, dehydrated, and proteolytically cleaved using trypsin. The peptide lysates were desalted using ZipTip-μC18 tips prior to nanoLC-MS/MS.

### Mass spectrometric measurement and data analysis

Peptide lysates were resuspended and injected into a nanoHPLC (UltiMate 3000 RSLCnano, Dionex, Thermo Fisher Scientific). The samples were then trapped in a C18 reverse-phase trapping column. The peptide lysates were separated by a C18 reverse-phase analytical column (Supplementary Methods). Subsequent mass spectrometric analysis of the eluted peptides was performed on a Q Exactive HF mass spectrometer (Thermo Fisher Scientific, Waltham, MA, USA) coupled with a TriVersa NanoMate ion source (Advion, Ltd, Harlow, UK) in LC chip coupling mode. The ionization and mass spectrometric settings are described elsewhere [45]. The acquired MS/MS spectra were searched against the constructed fungal database using the “Sequest HT” search algorithm (Proteome Discoverer, v2.5; Thermo Scientific). The constructed database included data from 90 fungal genomes differentiated into 84 fungal taxa representing 50 fungal genera (Supplementary File 1). Database selection was based on the most abundant species identified from the tailored amplicon sequencing experiment, their presence in a previous study conducted in the same geographical region [11], and our knowledge of common wood-associated fruiting bodies in these temperate forests [39]. Our database also included protein data from contaminants, tree species (UniProt release August 2021), and the most abundant bacteria (UniProt release January 2021) (Supplementary File 2). Data processing was performed using “Percolator” to assign reliable statistical confidence values [46] and “Pout2Prot” for protein grouping [47] exclusively of proteins assigned to fungi and prokaryotes. The resulting list of protein groups was imported into “Prophane” [48] for taxonomic annotation against the NCBI database and functional annotation against EggNOG (v5.0) and CAZy/dbCAN (Supplementary Methods). Protein group (PG) abundance quantification was performed by the normalized spectral abundance factor (NSAF) calculated from peptide spectral matches.

In total, 109 305 PGs were annotated, 97 490 of which were assigned to fungi and 11 476 to prokaryotes (Supplementary Table S3). Subsequent analyses exclusively involved PGs from fungi. The functional annotation focused on putative main extracellular enzymes involved in lignocellulolysis, which were categorized according to the CAZy database and their corresponding substrates [23]. These included enzymes that act on “lignin and related/derived aromatics” (laccase AA1 and several class-II peroxidases (PODs), AA2; unspecific peroxygenase, UPO; dye-decolorizing peroxidase, DyP); enzymes that are “lignocellulose associated” (i.e. H_2_O_2_-producing oxidoreductases, mostly oxidases of the CAZyme class AA3: (aryl) alcohol oxidase (AOs), pyranose 2-oxidase); enzymes that attack “cellulose” (cellobiose dehydrogenase (CDH), β-glucosidase GH3, and GH5_22; cellobiohydrolase (CBH): GH6 and GH7; endoglucanase: GH5_5 and GH45; LPMO: AAs 9, 10, and 15); and “hemicelluloses,” such as xylan/glucomannan (β-xylosidase GH3, mannanase GH5_7, xylanase GH10, GH11, xyloglucanase GH74, acetyl xylan/feruloyl esterase CE1, and acetylesterase CE16) (Supplementary Table S2). These enzymes were cross-validated for their functional characteristics based on an inspection of their amino acid sequence and a neighbor-joining phylogenetic analysis (Supplementary File 2). Furthermore, the GH3 and GH5 enzymes were annotated and separated into subclasses using dbCAN3 [49] with default settings (Supplementary Methods).

### Data analysis and statistics

Data analysis, visualization, and statistical tests were performed in R (v4.2.2, [50]). The quality-controlled metaproteome data set included 50 fungal genera that were selected according to their abundance in the amplicon sequencing data set, which initially included 389 genera. To visualize the correspondence between the amplicon sequencing and metaproteomic data, a butterfly diagram was created using “ggplot2” [51], featuring the 50 most abundant overlapping fungal genera. Correlation analysis between the two methods was conducted using the function “mantel” (“vegan” package) [52]. Furthermore, an analysis of the potential impact of tree species, tree clade (angio- vs. gymnosperms), and forest stand (i.e. plot) on community composition was tested for both methods by a three-way permutational multivariate analysis of variance (three-way PERMANOVA) based on Bray–Curtis distance using the function “adonis2” (“vegan” package) [52]*.*

Subsequent steps analyzed particular wood-inhabiting fungal genera in lignocellulose degradation, and therefore NSAFs of the PGs were log10-transformed. To identify the decomposing fungi and their enzymes, we created heatmaps with “ggplot2” to visualize the taxonomic assignment at the genus level, the involved tree species, and their corresponding decomposition functions. Detailed insights into the relevant correlations between enzymes and substrate types are illustrated in scatter plots (“ggplot2”). The relationships between substrate-specific enzymes were analyzed using the “lm” function (“stats” package). Previously, the normality of the distribution of variables was assessed using histograms (“hist” function) for the variables and QQ plots (“ggqqplot” function) for the model residuals in the “ggpubr” package.

To investigate the relationship between mass loss or species richness and CAZyme abundance (NSAF), we used a linear mixed effect model (LMM) with tree species as a random factor. Models were fitted with the “lme4” package for all the summarized CAZymes and substrate-specific enzymes, specifically those that act on lignin, are lignocellulose associated (H_2_O_2_-producing) or attack cellulose and hemicelluloses [53]. The quantitative predictors are scaled to zero mean and associated variance to allow comparison of estimates. The resulting estimates were subsequently visualized, and the predicted slopes, calculated with the “predict” function, were plotted against the raw data points. Normal distributions were checked by visual inspection with a QQ plot using the “ggqqplot” function (“ggpubr” package).

The functional redundancy was calculated with respect to fungal lignocellulose degradation. These were assessed based on the abundance-based fungal functional dissimilarities using Bray–Curtis dissimilarity. Using the “uniqueness” function (“adiv” package) [54, 55], the number of fungal species (N), Simpson index (D), Rao diversity (Q), and functional redundancy (R) were calculated. The scale of functional redundancy ranges from 0 (no redundancy) to 1 (high redundancy). For visualization, boxplots were generated based on tree species and substrate-specific enzyme types per tree species. An ANOVA was performed to examine the influence of tree clade and species on community-level functional redundancy.

## Results

### Comparison of amplicon sequencing and metaproteomic data

Using amplicon sequencing, 1184 OTUs were obtained: 757 (63.9%) belonged to Ascomycota, 316 (26.7%) belonged to Basidiomycota, and the remainder belonged to other or unknown fungal taxa (Supplementary Table S3). A total of 389 fungal genera were identified. The most abundant genus was *Cadophora* (5.4%; Ascomycota, putative endophytes/parasites), followed by *Ischnoderma* (5.1%; basidiomycetous WRF), *Ascocoryne* (4.3%; ascomycetous endophytic wood saprotroph), and several other wood-rot fungi (Supplementary Fig. S1). A PERMANOVA for amplicon sequencing analysis revealed a strong tree species effect (*P* = .001) and strong differences between tree clades (*P* = .001) but no plot effect (*P* = .332) for the fungal OTU-based community (Supplementary Table S5).

Metaproteomics revealed more than 223 000 unique peptides, subsequently used for parsimony-principle protein grouping. Leveraging the respective fungal database, we annotated 97 490 fungal PGs (Supplementary Table S3). Our findings indicate that *Armillaria* (basidiomycetous WRF) was the most abundant genus, representing 22.6% of the total protein abundance. It was followed by typical wood-decomposing fungal genera, such as *Fomitopsis* (5.2%, BRF), *Hypholoma* (4.9%, WRF), *Mycena* (3.9%, WRF), *Ischnoderma* (3.8%, WRF), *Antrodia* (3.6%, BRF), *Resinicium* (3.6%, WRF), *Trametes* (3.3%, WRF), and the abundant xylariaceous SRF *Annulohypoxylon* (2.5%) (Supplementary Fig. S1). Overall, 67.4% of the total protein abundance was related to WRF, 8.8% to BRF, 8.5% to SRF, and the remaining 15.3% to wood-associated endophytic and saprotrophic fungi (Supplementary Fig. S1).

The rank-abundance of the metaproteomic approach slightly differed from the amplicon sequencing approach (Supplementary Fig. S1). However, a Mantel test revealed a significant correlation (*P* < .001) in the mean abundance of fungal genera among the decomposing tree species (Supplementary Table S4 and Supplementary Fig. S2) but not in the comparisons of all single-log samples. Distinct differences were observed for *Armillaria*, which had a correspondingly low OTU frequency of 0.08%, and several genera that remained undetected via amplicon sequencing (Supplementary Fig. S1). In contrast, *Cadophora* had an amplicon frequency of 5.4%, compared with a 0.4% metaproteome abundance (Supplementary Fig. S1).

### Detection of fungal lignocellulolytic CAZymes

**Figure 1:**
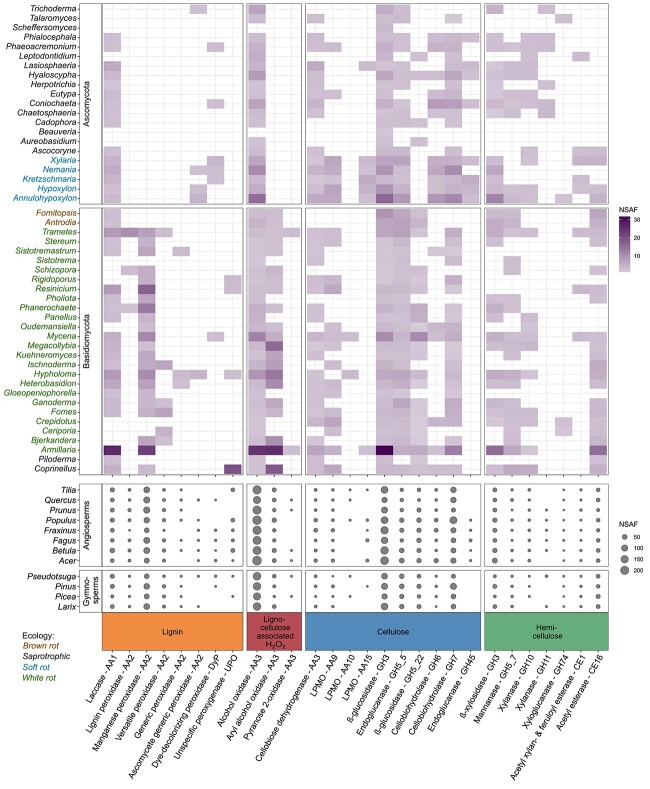
Heatmap showing the mean NSAF of selected fungal CAZymes that act on lignin and/or other aromatics, cellulose, and hemicelluloses or exhibit supportive activities related to lignocellulose modification and degradation (lignocellulose-associated, H_2_O_2_-forming); modified according to Reference [94]. Fungal CAZymes are related to each fungal genus and ecology according to our underlying genome database. Additionally, they are linked to decomposing trees (logs) categorized into angiosperms and gymnosperms.

Overall, 49 fungal PGs accounting for 2.6% of the total relative abundance were linked to the main lignocellulolytic CAZymes (i.e. Supplementary Table S2), of which *Armillaria* was the most abundant, with a 0.6% relative abundance (Supplementary Figs S1 and S3). For all the fungal genera, lignocellulolytic CAZymes were assigned based on their activity toward lignin, cellulose, hemicelluloses, or associated functions (Fig. 1, Supplementary Table S2).

**Figure 2:**
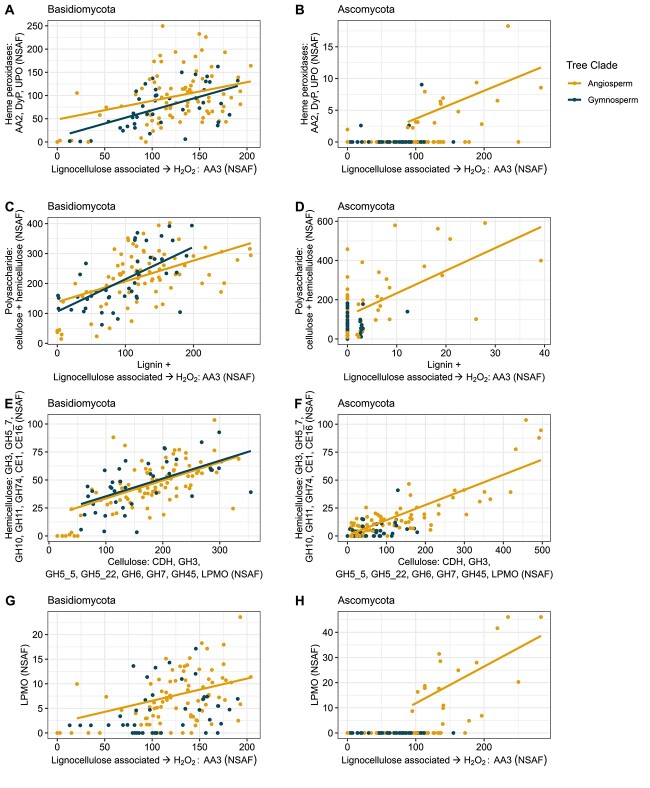
Correlations of lignocellulolytic CAZymes (i.e. ligninolytic class-II PODs/AA2 and other peroxidases, H_2_O_2_-generating oxidoreductases/AA3, hemicellulolytic and cellulolytic GHs and LPMOs) with their target substrates. The data points represent the cumulative NSAF of the different enzymes per sample separated at the fungal phylum level (Ascomycota and Basidiomycota) and according to occurrence in logs of angiosperms and gymnosperms. The significant regressions (*P* < .05) are displayed, whereas nonsignificant regressions are omitted from the plot.

The spectra of enzymes from Ascomycota and Basidiomycota exhibited distinct differences (Fig. 1). High-redox potential ligninolytic class-II PODs originated exclusively from basidiomycetous WRF and included mainly manganese peroxidase (MnP) and, to a lesser extent, versatile peroxidase (VP) and lignin peroxidase (LiP) (Fig. 1). Many peptides of lignin-modifying laccases derived from both fungal phyla were observed in the decaying logs. Peptides of DyPs and UPOs, which systematically belong to their own protein (super)families, were less frequently detected (Fig. 1). Peroxide-supplying enzymes of the CAZy group AA3 were also found (Fig. 1), namely, various AOs from both phyla and aryl-alcohol oxidase (AAO), which is specific for Basidiomycota. Pyranose 2-oxidases (i.e. glucose oxidase) play only a minor role in this process (Fig. 1). Key enzymes involved in cellulose decomposition and subsequent metabolism came from Asco- and Basidiomycota and included β-glucosidase (GH3, including dbCAN-defined subtypes e1, e16, e32, e51, e116, e147, e180, and e223; GH5_22), CBH (GH7), and endoglucanase (GH5_5), as well as, in lower abundance, CBH from the family GH6, oxidoreductase LPMO (AA9), and CDH (AA3). LPMOs from families AA10 and AA15 and ascomycetous endoglucanases from family GH45 were identified to a lesser extent (Fig. 1). The specific hemicellulolytic enzymes included frequently β-xylosidase (GH3, including subtypes e0, e21, e73, and e88), xylanases (especially the GH10 family), mannanase (GH5_7), and acetyl esterase (CE16) from Ascomycota and Basidiomycota. In addition, xyloglucanase (GH74) and acetyl xylan/feruloyl esterase (CE1) were less frequently observed (Fig. 1).

PERMANOVA revealed a strong tree species effect (*P* = .001) and plot effect (*P* = .012) for the fungal genera linked to lignocellulolytic CAZymes (Supplementary Table S5, Supplementary Fig. S3). However, considering the principal function of maintaining lignocellulolysis, it became evident that all the main extracellular enzymes were present in each tree species (Figs 1 and S4).

### CAZyme correlations in angiosperm and gymnosperm wood

Correlative analyses provided insights into the interplay of different substrate-dependent enzyme types among Ascomycota and Basidiomycota and between angiosperms and gymnosperm logs (Fig. 2). Many of the analyzed enzyme (sub)classes and individual enzyme types exhibited strong simultaneity (Fig. 2). Specifically, enzyme correlations for Basidiomycota occurred in angio- and gymnosperm logs, whereas for (mostly xylariaceous) Ascomycota, they appeared exclusively in angiosperm logs (Figs 2 and S6–S13).

A significant correlation with high abundance was detected between peroxide-producing enzymes (oxidases, AA3) and ligninolytic heme peroxidases in Basidiomycota (Fig. 2A and B), highlighting the interrelated action of these enzyme types during wood decay. However, ascomycetous heme peroxidases (i.e. low-redox potential generic class-II PODs, DyPs, and UPOs) were only moderately abundant in the angiosperm samples (up to 18 cumulative NSAF compared with up to 250 NSAF for Basidiomycota; Fig. 2A and B). Generally, lignin-modifying enzymes, including peroxide-providing oxidases (AA3), correlated significantly positively with enzymes responsible for polysaccharide degradation (i.e. cellulose and hemicelluloses) (Fig. 2C and D). A comparison of cellulolytic and hemicellulolytic enzymes revealed significant positive correlations (Fig. 2E and F). Furthermore, significant correlations of peroxide-providing enzymes (AA3) with LPMOs were observed for Ascomycota and Basidiomycota only in angiosperm logs (Fig. 2G and H), indicating a distinct role of LPMOs during wood decay.

The contribution of each fungal genus differed, especially when comparing the logs per tree clade (Supplementary Figs S6–S13). Among the Basidiomycota, the genera *Armillaria*, *Hypholoma*, and *Resinicium* exhibited considerable ligninolytic potential because of the significant correlations between H_2_O_2_-delivering enzymes and high-redox potential peroxidases (MnP, VP, LiP) in angiosperm and gymnosperm logs (Supplementary Fig. S6). *Bjerkandera*, *Coprinellus*, *Fomes*, *Ganoderma*, *Ischnoderma*, and *Trametes* exhibited similar patterns exclusively in angiosperm logs and *Heterobasidion* in gymnosperm logs (Supplementary Fig. S6). All abovementioned fungi possess the necessary enzymes to substantially degrade cell wall polysaccharides, as do *Antrodia* and *Fomitopsis* (both BRF) in gymnosperm logs (Supplementary Figs S8 and S10). Within Ascomycota, mainly *Annulohypoxylon*, *Hypoxylon*, *Kretzschmaria*, *Nemannia,* and *Xylaria,* exhibited high enzyme abundances and significant correlations between different enzyme types. These genera are almost exclusively found in angiosperms and belong to the order Xylariales, which are classified as SRF (Supplementary Figs S7, S9, S11, and S13).

### Effects of mass loss and species richness on the metaproteome

The estimated mass loss, ranging from 17.1% to 78.1%, was lowest in *Fraxinus* (34 ± 11%) and highest in *Betula* (67 ± 8%) and *Fagus* (65 ± 3%) (Supplementary Fig. S5), which was reflected by a significant tree species effect (*P* < .001, Supplementary Table S6). In general, the gymnosperms (46 ± 12%) exhibited lower mass loss than the angiosperms (59 ± 8.5%, *P* < .001; Supplementary Table S6) after 8 ½ years of decomposition. However, no significant correlations were found between the total CAZymes or between CAZymes separated according to their substrates and the estimated mass loss as a decay proxy if the tree species were considered (Supplementary Table S6). These correlations varied distinctly among the tree species, revealing both positive and negative directions (Supplementary Fig. S5, Supplementary Table S6). Only considering all samples irrespective of tree species, a significant positive correlation was observed between mass loss and all the CAZymes (*P* = .0053; Supplementary Table S6, Supplementary Fig. S5).

**Figure 3:**
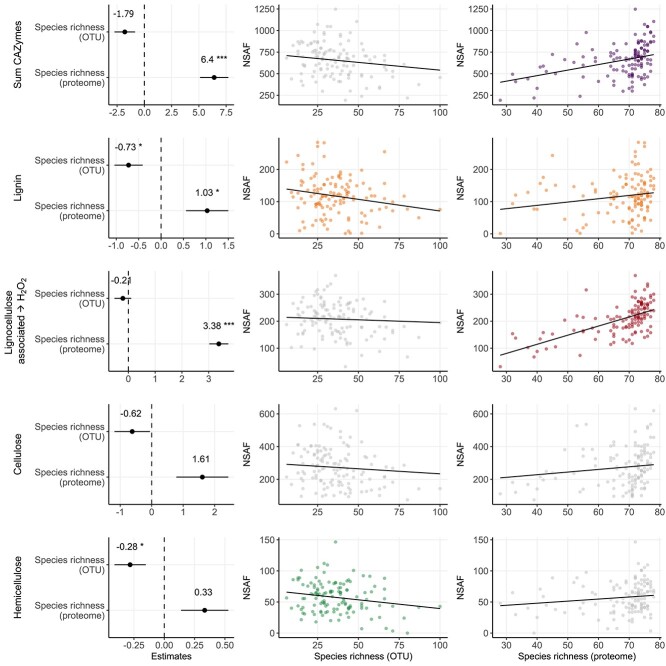
Effects of fungal amplicon sequencing- and metaproteomics-based species richness on the CAZymes (NSAF) and substrate-specific subgroups. Lines represent fits of the linear mixed-effects model with tree species as a random factor. Significant relationships (*P* < .05) are shown as colored points, whereas grey points indicate no significant relationships. See Supplementary Table S8 for details on the fitted models.

OTU-based species richness ranged from 6 to 100 per sample and was lowest in *Tilia* (21 ± 7) and highest in *Picea* (56 ± 25). The metaproteome-based species richness ranged from 28 to 78, with the lowest occurring in *Quercus* (56 ± 16) and the highest occurring in *Populus* (75 ± 3). For both approaches, species richness exhibited significant tree species effects (OTU: *P* < .001, proteome: *P* = .004; Supplementary Table S7), but no significant differences were detected among the plots (OTU: *P* = .076, proteome: *P* = .319; Supplementary Table S7). When the species richness of both approaches was compared against that of the lignocellulolytic metaproteome (i.e. CAZyme NSAF), an opposite trend was observed. The OTU-based species richness was negatively correlated (Fig. 3), particularly significantly for ligninolytic and hemicellulolytic enzymes (*P* < .05; Supplementary Table S8). The metaproteome-based enzyme-producing species richness (counted based on the 84 taxa in the underlying database) was positively correlated with the enzyme abundance (Fig. 3) and was significant for all CAZymes (*P* < .001), ligninolytic enzymes (*P* < .05), and peroxide-producing enzymes (*P* < .001; Supplementary Table S8).

### Functional redundancy

**Figure 4:**
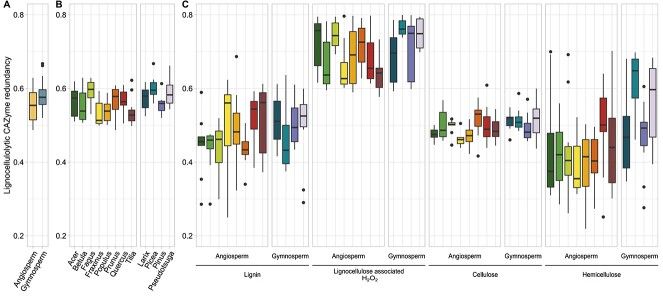
The functional redundancy of lignocellulolytic CAZymes was assessed across tree clades (A), tree species (B), and separately by substrate-specific enzyme classes (C). The data were processed according to Ricotta and Pavoine [55], and the community-level functional redundancy was calculated. For detailed statistical analysis, see Supplementary Table S9.

The presence and high abundance of many main lignocellulolytic enzymes (e.g. laccase, MnP, AOs, endoglucanase, CBH, and β-glucosidase) in all the tree species indicated functional redundancy (Fig. 1). Generally, a high functional redundancy of all lignocellulolytic CAZymes was observed across the 12 tree species (Fig. 4); it was lowest in *Fraxinus* (0.53 ± 0.04) and highest in *Picea* (0.62 ± 0.04), resulting in a significant tree species effect (*P* < .001) (Supplementary Table S9). Gymnosperm logs generally exhibited slightly greater redundancy (0.60 ± 0.04) than angiosperm logs (0.57 ± 0.06, *P* < .01; Fig. 4). However, distinct differences were noted considering the enzyme substrates, with the highest redundancy for enzymes that provide peroxide (AA3) (0.72 ± 0.08) as a cosubstrate for various other enzymes (PODs, DyPs, UPOs, LPMOs) as well as for Fenton chemistry, followed by enzymes that are active on cellulose, specifically diverse GHs and LPMOs (0.52 ± 0.04), and followed by lignin-modifying enzymes, specifically laccases and peroxidases (0.48 ± 0.1) and hemicellulolytic enzymes (GHs and CEs, 0.45 ± 0.13) (Fig. 4).

## Discussion

### Key fungi and lignocellulolytic enzymes

This study provides detailed insight into the fungal deadwood community and its enzymatic machinery that causes a complete breakdown of the lignocellulosic complex. The basis is the *a priori* knowledge of the fungal community composition at the molecular and fruiting body levels from the BELongDead Experiment [11, 28, 36, 39]. In addition, the constant inflow of new fungal genomes into databases derived from large genome-sequencing projects such as DOE-JGI Mycocosm [56] and our own sequencing efforts over the past decade [57-59] has provided the necessary data for this broad metaproteomic approach. Our database-dependent metaproteomic approach relies on amplicon sequencing results and is remarkably consistent and correlative (e.g. the Mantel test results). With both approaches, we see strong tree species effects, as shown in previous amplicon sequencing studies [11, 28, 36], and large differences between the rotting process in angiosperm and gymnosperm logs. This finding supports the assumption that most fungi are adapted to specific substrates, except for ubiquitous generalists such as the honey mushrooms *Armillaria* [60]. Generally, the analyzed metaproteome was dominated by Basidiomycota (accounting for ~67% of the identified fungal peptides), specifically WRF, although more Ascomycota taxa were included in the underlying proteome database. The predominant fungal genus *Armillaria* comprises notorious tree parasites (e.g. those causing root rot) and powerful wood decomposers, known to harbor one of the largest and oldest organisms on earth [60-63]. The next most abundant genera (e.g. *Fomitopsis*, *Hypholoma*, *Mycena*, *Ischnoderma*, *Antrodia*, *Resinicium*, *Trametes*, *Annulohypoxylon*) are also well-known wood-rot fungi and are widely distributed across temperate forests in Eurasia and North America [6, 11, 64, 65].

In addition, we gained detailed information about their function, including the enzymes they secrete to digest and process polymeric wood components and their preferred target substrates (angiosperm vs. gymnosperm wood, cellulose vs. hemicelluloses vs. lignin vs. low-molecular-mass fragments and wood ingredients). Basidiomycetous MnP was the most prominent and abundant ligninolytic enzyme [13, 66] found in all decaying tree species in this study and was derived from diverse WRF species. This was expected, although not to this extent, as previous detections of MnP activities (via oxidation of Mn^2+^ into Mn^3+^) in field samples have been highly variable and scattered compared with measurements of other enzymes such as laccase or GHs [67, 68]. Thus, these results confirm the key role of MnP in lignin degradation by Basidiomycota, as predicted by previous field studies and laboratory experiments with fungal cultures or isolated enzymes [11, 67, 69-71]. Laccases, which have a polymerizing (coupling of phenolics) and, under certain circumstances (i.e. in the presence of suitable redox mediators), depolymerizing effect on lignin fragments, are frequently observed during wood decomposition [67, 72, 73]. Laccases, which generally have lower redox potentials than peroxidases (<0.82 V vs. 0.9–1.5 V), were highly abundant in almost all the wood samples in our study and originated from a wide variety of Basidiomycota, but also from many Ascomycota (primarily Xylariales but also Helotiales and Sordariales). Reportedly, laccases contribute to the detoxification of phenolic compounds present in wood or released during lignin decay, which may explain their widespread occurrence and use by both Ascomycota and Basidiomycota [13, 74]. Two other ligninolytic class-II PODs (AA2) of Basidiomycota, namely, LiP and VP, were also present in all the logs; however, they were found in much lower abundances than MnP and originated exclusively from a few basidiomycetous genera (e.g. LiP: *Trametes*, *Phanerochaete, Schizopora*; VP: *Bjerkandera*, *Fomes*, *Ischnoderma*), which has already been indicated in several genomic studies [15, 17]. Other ligninolytic enzymes, such as generic peroxidase, DyP, and UPO, were infrequently detected and seemed to play only minor roles.

The most important enzymes for cellulose degradation were endocellulases and CBHs (GH5_5, GH6, and GH7), LPMOs (mainly from AA9), CDH (AA3), and β-glucosidase (GH3 and GH5_22), which are secreted in large quantities, ultimately providing glucose that is metabolized by the Ascomycota and Basidiomycota fungal communities. Furthermore, hemicellulolytic enzymes such as xylanase (GH10, GH11), β-xylosidase (GH3), mannanase (GH5_7), xyloglucanase (GH74), and esterases were present (CE1, CE16), with GH3 and CE16 being the most predominant enzymes from Ascomycota and Basidiomycota, eventually delivering other monosaccharides (xylose, mannose) derived from the backbone of diverse hemicellulose constituents. The importance of these detected cellulolytic and hemicellulolytic enzymes is well known from larger genome-sequencing projects and multiple laboratory studies of Ascomycota and Basidiomycota, and these enzymes were also found in deadwood field experiments [6, 11, 17]. Peroxide-producing AA3 oxidoreductases such as AOs and basidiomycetous AAO were the most abundant enzyme groups in this study and were present in almost all the logs. Along with other enzyme types, these enzymes were strongly positively correlated with ligninolytic peroxidases (for which they provide the necessary peroxide as a cosubstrate) as well as with ascomycetous and basidiomycetous LPMOs. LPMOs are copper-containing biocatalysts that can act as monooxygenases and peroxygenases [75, 76]. These enzymes are thought to play a crucial role in the initial attack of crystalline cellulose by oxidatively cleaving glycosidic bonds to form amorphic moieties, allowing hydrolytic cellulases to break down biomass more efficiently [76]. LPMOs were shown to interact with CDH, a flavin and heme-containing enzyme that oxidizes sugars (e.g. cellobiose) to the corresponding lactones (thereby removing excess cellobiose and avoiding negative feedback loops) [77] while donating the abstracted electrons directly to partner enzymes (e.g. LPMOs whose active site copper is reduced; Cu^2+^ ➔ Cu^+^) or generating H_2_O_2_ by reducing dioxygen (O_2_) [78-80]. Thus, CDH and AA3-type oxidases (AOs, pyranose oxidase) may fuel LPMOs with H_2_O_2_ and thus force the peroxygenase mode [81]. LPMOs were found along with CDH in all decomposing log species, and in addition to Basidiomycota, they particularly originated from the xylariaceous Ascomycota. In addition, CDH was postulated to be involved in complex redox cycling and Fenton chemistry, during which hydroxyl radicals (^•^OH) are formed; these radicals are the most potent oxidants in biological systems, and they are also active during lignocellulolysis.

Taken together, the results of this metaproteomic approach highlight wood-rot Basidiomycota as the main driver of wood decay, although Ascomycota, especially Xylariales, may also play a relevant role, except in substantial lignin decomposition. It is known from the literature that lignin is less mineralized during the rotting process by *Xylaria* (<10% degradation to CO_2_, compared with up to 75% lignin mineralization by basidiomycetous WRF) [71] and rather released in the form of large water-soluble lignin-polysaccharide fragments [22]. This study revealed the strong simultaneity of the different substrate-acting enzyme types and, further, the strong interrelatedness of the enzymes when peroxide-producing and peroxide-using enzymes were considered. However, it is essential to exercise caution and bear in mind that this approach used ~5 g of wood collected across the entire log diameter. Therefore, enzymes may be present at different spots and scales and originate from different fungal organisms in decomposing wood.

### Role of mass loss and species richness

The estimated mass loss after ~8 ½ years is primarily tree species dependent, as shown in several previous studies [10, 35, 82]. However, across the 12 tree species, we did not observe a significant relationship between mass loss and the metaproteome for the CAZymes or the substrate-specific enzyme subgroups. We could only observe a trend toward increased CAZyme abundance with increasing mass loss by analyzing all samples independent of the tree species. However, our sampling approach prevents larger generalization and needs to be further tested with replicated measurements over time of the same decomposing logs and in a wider geographical setting. Nevertheless, the ubiquitous presence of diverse CAZymes already indicates substantial functional redundancy across a wide range of mass losses as a proxy for different decay stages. This was supported by the observed high enzymatic activities during several decay stages in three different tree species in another study [6].

In this study, species richness was not correlated with mass loss, but strong correlations with CAZymes or substrate-specific subgroups were observed. The OTU-based species richness was negatively related to the metaproteome, indicating increasing CAZyme abundance with decreasing fungal species. Similar negative correlations were observed for ligninolytic [83] and (hemi)cellulolytic enzymes (both indicative of microbial activity) as well as for fungal biomass [11]. Furthermore, negative correlations between richness and mass loss were ascertained in laboratory experiments, indicating greater competition when multiple species are involved in the decay process [84]. However, positive correlations have also been observed under near-natural conditions [85] and are known for other ecosystem processes [86]. In this study, a comparable positive correlation was observed between the metaproteomic-calculated active fungal species richness and the abundance of CAZymes or substrate-related subgroups. In deadwood, a similar positive relationship was revealed for fungal fruiting body richness indicating active species and microbial respiration [87]. Although our findings may include autocorrelated behavior because of the use of a selected database for deadwood-degrading fungi, the results should be compared with other approaches covering the active fungal community, such as transcriptomic methods. Nevertheless, our study suggested that a higher richness of active fungal species corresponds to greater amounts of CAZymes and consequently a higher microbial activity. The contrasting results between our two approaches, amplicon sequencing and metaproteomics, are certainly related to the fact that DNA-based techniques capture inactive or dead species and consider a broader range of species than metaproteomics because of current data limitations. DNA-based techniques might also be biased when transcriptional activity is considered. Future improvements to genomic and proteomic databases and larger sample sizes will improve our knowledge.

### Functional redundancy

Microbial functional redundancy is a widely recognized mechanism for understanding ecosystem processes but always depends on the considered functions [31, 88]. During deadwood decomposition, WRF and BRF, and to some extent SRF, are considered the main drivers, and it seems that replacing, changing, or manipulating this community has little effect on this process (e.g. [89], general concepts in Reference [31]). Often, the entire potential metabolic functions based on metagenomes or functional assignments of OTUs via databases serve as a basis for revealing functional redundancy [90]. In our study, we focused on the truly active fraction by analyzing the present lignocellulolytic enzymes and their fungal origin. Across different decomposing logs, we found rather high functional redundancy for lignocellulose degradation, which was certainly driven by key enzymes such as MnP, AOs, and several GHs. Considering the strong tree species effect and resulting specialization of the fungal community, the core community likely contains similar redundant enzymes. There are still several limitations: (i) different enzymes of the same CAZy family or with identical EC numbers nevertheless have different physicochemical and kinetic properties (e.g. in terms of substrate affinity, reaction rate, and catalytic efficiency), (ii) the detected enzymes could be readily inactivated, and (iii) polysaccharide hydrolases often show side activities toward other substrates or whose actual activity is not yet known. However, the key enzymes of this study have well-documented activities and have been characterized in laboratory studies [23]. The highest redundancy was observed for peroxide-producing (AA2) enzymes, but since they supply peroxidases and LPMOs with their cosubstrate (H_2_O_2_) and the latter is involved in the formation of reactive oxygen species (radicals), these findings have a plausible background. Other enzymes, which may be more context specific and used only occasionally, exhibit activities that are highly spatially variable in field studies [91].

From a conceptual point of view, the question arises whether lignocellulolysis in deadwood is a narrow or a broad functional process [31]. Given the large number of microbes (filamentous fungi, yeasts, protists, bacteria, and archaea) that benefit from wood decomposition and the fact that substantial lignocellulolysis is mainly carried out by only a few specialized fungi, this process is rather narrow. However, this statement is debatable because lignocellulolysis still involves hundreds of adapted fungal taxa from different fungal groups with considerable niche widths and competitive capabilities [92] and also relies on other taxonomic groups involved in deadwood fragmentation (e.g. arthropods) or nutrient acquisition (e.g. nitrogen-fixing bacteria), which broadens this process and likely makes it more resilient to potential disturbance. This *in situ* study elucidated the molecular processes related to fungi during wood decomposition, enabling us to address future questions about changing environmental factors and the resulting relevance and resilience of ecosystem functions, such as lignocellulolysis, to sustain the health and stability of ecosystems in the face of ongoing anthropogenic changes [93].

## Supplementary Material

supplementary_material_wrae050

## Data Availability

Mass spectrometry-based metaproteomic data are available at PRoteomics IDEntifications (PRIDE) under accession number PXD041962 (https://www.ebi.ac.uk/pride/). The experimental metadata (DOI: 10.1038/s41467-021-26 111-3) were generated using lesSDRF (DOI: 10.1038/s41467-023-42 543-5) and are also available on PRIDE. Annotated metaproteomic data and amplicon OTU tables are available at BExIS under accession numbers 31417 for metaproteomic and 31456 for amplicon sequencing (https://www.bexis.uni-jena.de/). The mass loss data are available under accession number 27126 in BExIS. Raw amplicon sequencing data were submitted in the Short Read Archive (NCBI SRA) under BioProject number PRJNA756463 (SAMN20956281–SAMN20956430).
