# Supplementary material for: Enzymatic machinery of wood-inhabiting fungi that degrade temperate tree species
Source: ISME J. 2024 Mar 22;18(1):wrae050. doi: 10.1093/ismejo/wrae050 (PMC11022342; doi:10.1093/ismejo/wrae050)
Supplement: supplementary_material_wrae050 [file supplementary_material_wrae050.zip › 20240313_Supplements_resubmissionv15_FINAL.docx]

**Supplementary information**

**Enzymatic machinery of wood-inhabiting fungi that degrade temperate tree species**

Authors: Lydia Kipping^1,2^, Nico Jehmlich^1^, Julia Moll^3^, Matthias Noll^2,4^, Martin M. Gossner^5,6^, Tim Van Den Bossche^7,8^, Pascal Edelmann^9^, Werner Borken^4^, Martin Hofrichter^10^, Harald Kellner^10^*****

^1^Helmholtz-Centre for Environmental Research - UFZ GmbH, Department of Toxicology, Leipzig, Germany

^2^Institute for Bioanalysis, University of Applied Sciences Coburg, Coburg, Germany

^3^Helmholtz Centre for Environmental Research—UFZ GmbH, Department of Soil Ecology, Halle, Germany

^4^Bayreuth Center of Ecology and Environmental Research (BayCEER), University of Bayreuth, Bayreuth, German

^5^Forest Entomology, Swiss Federal Research Institute WSL, Birmensdorf, Switzerland

^6^Department of Environmental Systems Science, Institute of Terrestrial Ecosystems, ETH Zürich, Zürich, Switzerland

^7^VIB - UGent Center for Medical Biotechnology, VIB, Ghent, Belgium

^8^Department of Biomolecular Medicine, Faculty of Medicine and Health Sciences, Ghent University, Ghent, Belgium

^9^Department of Ecology and Ecosystem Management, Center of School of Life and Food Sciences Weihenstephan, TU München, Freising, Germany

^10^Department of Bio- and Environmental Sciences, International Institute Zittau, TU Dresden, Zittau, Germany

*** Correspondence: Harald Kellner, E-Mail: harald.kellner@tu-dresden.de**

**Supplements:**

- Supplementary methods
- 9 Supplementary tables
- 13 Supplementary figures

1. **Supplementary methods**

**Protein extraction and sample preparation for mass spectrometric analysis**

The collected wood chips were handled according to a previously described procedure [44]. Initially, the wood chips were roughly sliced with a blade and then homogenized to a fine powder with an analytical mill (IKA A11 basic, IKA grinder) using liquid nitrogen. 5 g of wood powder were transferred into 50 mL tubes for cell lysis with 20 mL sodium dodecyl sulfate (SDS) buffer (5% (w/v) SDS, 20 mM DTT, 100 mM Tris). After rotation overnight, three thaw-freeze cycles and ultrasonication (UP50H, Hielscher, Teltow, Germany) were applied. Next, the supernatant was collected for protein extraction, and 5 mL phenolic extraction buffer was added. The mixture was shaken for 30 minutes at room temperature, and the phases were separated by centrifugation (7,200 × g, 10 min, 4°C). After transferring the phenolic phase into a new 50 mL tube, the extraction procedure was repeated, and the resulting phenolic phases pooled in one tube. Extracted proteins were precipitated overnight at −20°C in precipitation solution (4-fold of 100 mM ammonium acetate in methanol) and harvested by centrifugation (7,200 × g, 10 min, 4°C). The remaining protein pellets were washed three times with 1 mL precipitation solution and finally once with 1 mL ice-cold acetone. Then, the dried pellets were dissolved in SDS sample buffer (2% w/v SDS, 2 mM β-mercaptoethanol, 4% v/v glycerol, 40 mM Tris–HCl pH 6.8, 0.01% (w/v) bromophenol blue), heated to 90°C for 4 min and separated by SDS polyacrylamide gel electrophoresis. The protein bands were cut from the gel, destained, dehydrated, and proteolytically cleaved with trypsin (Promega) at 37°C overnight. The digested peptides were extracted and desalted using ZipTip-μC18 tips (Merck Millipore, Darmstadt, Germany).

**Mass spectrometric measurement**

Peptide lysates were resuspended in 0.1% formic acid prior to injection into nanoHPLC (UltiMate 3000 RSLCnano, Dionex, Thermo Fisher Scientific). First, 5 µL lysate was trapped in a C18 reverse-phase trapping column (C18 PepMap100, 300 µm × 5 mm, particle size 3 µm, Thermo Fischer Scientific) for 5 min at 4% mobile phase B (80% acetonitrile in nanopure water with 0.08% formic acid) and 96% mobile phase A (nanopure water with 0.1% formic acid). Then, the peptides were separated by a C18 reverse-phase analytical column (Acclaim PepMap^®^ 100, 75 µm × 25 cm, particle size 3 µm, nanoViper, Thermo Fischer Scientific) with a two-step gradient (90 min, 4% solvent B to 30% B, followed by 30 min from 30% B to 55% B; solvent A: 0.1% formic acid; solvent B: 80% acetonitrile, 0.1% formic acid) at 300 nL/min flow rate and a column temperature of 35°C [45]. Subsequent mass spectrometric analysis of the eluted peptides was performed on a Q Exactive HF mass spectrometer (Thermo Fisher Scientific, Waltham, MA, USA) coupled with a TriVersa NanoMate source (Advion, Ltd., Harlow, UK) in LC chip coupling mode. The ionization and mass spectrometric settings are previously described (see Supplementary section [45]).

**Metaproteomic data analysis**

The acquired MS/MS spectra were searched against the constructed fungal database using Sequest HT (Proteome Discoverer, v2.5; Thermo Scientific). To investigate wood-inhabiting and associated fungi, the constructed database included data from 90 fungal genomes (merged amino acid fasta file), differentiated into 84 fungal taxa representing 50 fungal genera (**Supplementary File 1**, “*Fasta-fungi_information.xlsx*”). The selection was based on the most abundant species identified from the tailored amplicon sequencing study, their presence in a previous study conducted in the same geographical region [11], and our knowledge of common wood-associated fruiting bodies in these temperate forests [39]. To improve annotation accuracy, our database incorporates protein data from contaminants, tree species (UniProt release August 2021), and the most abundant bacteria (UniProt release January 2021) (**Supplementary File 2**, “*Fasta_TaxonID_bacteria_tree-species.csv*”).

Search settings were as follows: enzyme specificity set for trypsin (Full) or Asp-N (Full) with up to two missed cleavages, a precursor mass tolerance of 10 ppm, and a fragment mass tolerance of 0.02 Da MS/MS. Carbamidomethylation of cysteines was specified as a fixed modification. False discovery rates (FDR) were determined using *Percolator* [46]. The proteins were filtered according to high confidence <0.01 FDR and further processed using *Pout2Prot* [47]. Therefore, we removed proteins from the list of identified proteins assigned to tree species and common laboratory contaminants such as keratins or trypsin. Only proteins assigned to fungi and prokaryotes were further processed in this study using *Pout2Prot* [47] to perform protein grouping. The output list was imported into the open-source software *Prophane* [48] for taxonomic and functional assignment of the protein groups. The protein groups (PG) were searched against the NCBI database for taxonomic annotation and the EggNOG (v5.0) and CAZy/dbCAN database for functional annotation. Annotations were based on the lowest common ancestor approach (setting 0.6) per protein group. The protein group abundance quantification was done by the normalized spectral abundance factor (NSAF) calculated from peptide spectral matches (PSMs).

In the study, 109,305 protein groups were annotated, 97,490 of which were assigned to fungi and 11,476 to prokaryotes (**Supplementary Table S3**). Since further analysis focused on deadwood-inhabiting and associated fungi, the dataset was separated and only used for fungi (97,490 protein groups) in all following analyses. The Prophane-based NCBI annotations were additionally checked against JGI (https://genome.jgi.doe.gov), and the taxonomy was manually edited if necessary. Functional annotation focused on putative main extracellular enzymes involved in lignocellulolysis (i.e., direct or indirect participation in the degradation and/or chemical modification of lignin, cellulose, hemicelluloses (mainly xylan, glucomannan)), which were categorized according to the CAZy database and the substrates they act on [23]. These included enzymes that act on “lignin and related/derived aromatics” (laccase AA1 and several class-II peroxidases PODs/AA2, unspecific peroxygenase UPO, dye-decolorizing peroxidase DyP), enzymes that are “lignocellulose associated” (i.e., H_2_O_2_-producing oxidoreductases, mostly oxidases of the CAZyme class AA3: (aryl) alcohol oxidase, pyranose 2-oxidase) as well as enzymes attacking “cellulose” (cellobiose dehydrogenase CDH, β-glucosidase GH3 and GH5_22, cellobiohydrolase GH6, GH7, endoglucanase GH5_5, and GH45, LPMO AAs 9, 10 and 15) and “hemicelluloses”, e.g. xylan/glucomannan (β-xylosidase GH3, mannanase GH5_7, xylanase GH10, GH11, xyloglucanase GH74, acetyl xylan/feruloyl esterase CE1, and acetylesterase CE16) (**Supplementary Table S2**). These enzymes, which were assigned to a protein group based on the *Prophane* search, were cross-validated for their functional characteristics based on inspection of their amino acid sequence and a NJ-phylogenetic analysis using Clustal Omega 1.2.2 [95] for alignment (**Supplementary File 2**, “*CAZymes. fasta*”). In addition, the GH3 and GH5 enzymes were also annotated and separated into subclasses using dbCAN3 [49] with default settings (dbCAN HMMdb v12, E-Value < 1e-15, coverage > 0.35).

1. **Supplementary tables**

*Supplement Table S1. Number of samples of 12 temperate tree species available in the field and number of final samples for subsequent analysis by metaproteomics and amplicon sequencing. Due to losses in laboratory handling and subsequent processing, a total of 127 wood samples were used for the analyses. Due to scarcity, Prunus included only eight replicates, whereas all other 11 tree species included 10 to 12 replicates each.*

| **Tree species** | **Logs available in the field** | **Samples with metaproteome result** |
| --- | --- | --- |
| Angiosperm: |  |  |
| *Acer* spp., maple | 11 | 10 |
| *Betula pendula* Roth, birch | 12 | 11 |
| *Fagus sylvatica* L., European beech | 12 | 10 |
| *Fraxinus excelsior* L., European ash | 11 | 11 |
| *Populus* spp., aspen | 12 | 12 |
| *Prunus avium* L., wild cherry | 8 | 8 |
| *Quercus* spp., oak | 12 | 11 |
| *Tilia* spp*.*, lime tree | 12 | 11 |
| Gymnosperm: |  |  |
| *Larix decidua* Mill., European larch | 11 | 10 |
| *Picea abies* L., H. Karst., Norway spruce | 12 | 10 |
| *Pinus sylvestris* L., Scots pine | 12 | 12 |
| *Pseudotsuga menziesii* (Mirb.), Franco, Douglas fir | 12 | 11 |
| **Sum** | **137** | **127** |

*Supplement Table S2. Classification and function of the analyzed lignocellulolytic CAZymes of deadwood-colonizing fungi (wood-rot and wood-associated fungi). *DyP and UPO have not yet been classified in the CAZy system, but were included because of their ability to cleave lignin model dimers and to oxidize other recalcitrant aromatics. Ligninolytic peroxidases, specifically MnP, LiP, and VP, occur exclusively in Basidiomycota, as do certain types of AAO. GH45 is only found in Ascomycota, while all other enzymes occur in Basidiomycota and Ascomycota. The GH3 family was phylogenetically and dbCAN3-based divided into β-1,4-glucosidase and β-1,4-xylosidase. The CAZy family GH5 was also separated into subfamilies using dbCAN3. CDH is not able to attack cellulose on its own but only in combination with LPMOs, which is why it is referred to as a CDH/LPMO system [77].*

| Enzyme names  ***(action on / activity towards)*** | EC numbers | CAZy families | Enzyme functions |
| --- | --- | --- | --- |
| ***Lignin and aromatics*** | | | |
| Laccase (*p*-diphenol oxidase) | 1.10.3.2 | AA1 | Oxidation of phenolic substrates with redox potentials E^0^ <0.8 V (incl. phenolic lignin subunits) using O_2_ as electron-accepting co-substrate, coupling of lignin fragments |
| Manganese peroxidase (MnP) | 1.11.1.13 | AA2 | Oxidation of Mn^2+^ into Mn^3+^ that is chelated by dicarboxylic acids and serves as lignin-oxidizing redox mediator (E^0^ 0.9-1,2 V); use of H_2_O_2_ as electron-accepting co-substrate; lignin breakdown (depolymerization and mineralization) |
| Lignin peroxidase (LiP) | 1.11.1.14 | AA2 | Oxidation of nonphenolic substrates (β-O-4 lignin subunits; E^0^ up to 1,5 V) using H_2_O_2_ as electron-accepting co-substrate; lignin breakdown (depolymerization) |
| Versatile peroxidase (VP) | 1.11.1.16 | AA2 | Functional hybrid of MnP, LiP, and GP; direct and indirect oxidation of phenolic and nonphenolic substrates (incl. lignin subunits) as well as of Mn^2+^ using H_2_O_2_ as electron-accepting co-substrate; lignin breakdown (depolymerization) |
| Generic peroxidase (GP) | 1.11.1.7 | AA2 | Oxidation of phenolic substrates (E^0^ <0.9 V) using H_2_O_2_ as electron-accepting co-substrate, coupling of lignin fragments |
| Dye-decolorizing peroxidase | 1.11.1.19 | DyP* | Oxidation of phenolic and nonphenolic substrates (incl. lignin dimers, E^0^ up to 1,4 V) and recalcitrant anthraquinone compounds (dyes) using H_2_O_2_ as co-substrate |
| Unspecific peroxygenase | 1.11.2.1 | UPO* | Oxidation and/or oxygenation of aromatic and aliphatic substrates (incl. lignin dimers, E^0^ up to 1,5 V), using H_2_O_2_ as co-substrate (electron acceptor and oxygen source), *O*-demethylation |
| ***Support of lignocellulose degradation (H_2_O_2_ production)*** | | | |
| Pyranose 2-oxidase | 1.1.3.10 | AA3 | Oxidation of pyranoses (e.g., D-glucose, D-Xylose) into corresponding sugar lactones (e.g., gluconolactone) and reduction of O_2_ to H_2_O_2_ |
| Aryl alcohol oxidase (AAO) | 1.1.3.7 | AA3 | Oxidation of aryl alcohols (e.g., benzyl alcohol, veratryl alcohol) into corresponding aromatic aldehydes and reduction of O_2_ to H_2_O_2_ |
| Alcohol oxidase (AO) | 1.1.3.13 | AA3 | Oxidation of primary alcohols (e.g., ethanol) into corresponding aliphatic aldehydes and reduction of O_2_ to H_2_O_2_ |
| ***Cellulose*** | | | |
| Cellobiose dehydrogenase (acceptor; CDH) | 1.1.99.18 | AA3 | Oxidation of cellobiose (or other sugars) into cellobionolactone (or other sugar lactones) and reduction of O_2_ (acceptor) to H_2_O_2_ or transfer of the electrons to other acceptors such as quinones, oxidized metal ions (e.g., Fe^3+^) or the active site of partner enzymes (e.g., Cu^2+^ in LPMOs – then referred as CDH/LPMO system [77]) |
| Lytic polysaccharide monooxygenases (LPMOs) | 1.14.99.54, 1.14.99.56 | AA9, 10, 15 | Oxidative cleavage of cellulose chains to fragments with either C1-lactone or C4-ketoaldose moieties at the reducing and non-reducing ends, respectively; use of O_2_ (monooxygenase mode, under delivering of electrons, e.g., from CDH) or H_2_O_2_ (electron acceptor & oxygen source; peroxygenase mode) |
| β-1,4-glucosidase, bi/multifunctional glycosidases | 3.2.1.21,  3.2.1.x | GH3 subtypes e1, e16, e32, e51, e116, e147, e180, e223 | Release of D-glucose units by hydrolysis of terminal β-1-4 glycosidic bonds from the non-reducing ends of cellulose chains (exocellulase activity), as well as fission of cellobiose |
| β-1,4-endoglucanase | 3.2.1.4 | GH5_5, GH5_22 | Hydrolytic cleavage of β-1-4 glycosidic bonds within cellulose chains in amorphic regions (endocellulase), beside CMCase activity GH5_22 also includes β-xylosidase activity [94-96] |
| cellobiohydrolase (non-reducing end) (CBH), also β-1,4-endoglucanase | 3.2.1.91,  3.2.1.4 | GH6 | Cleavage/release of cellobiose units from the non-reducing ends of cellulose chains (exocellulase); hydrolytic cleavage of β-1-4 glycosidic bonds within cellulose chains in amorphic regions (endocellulase) |
| Cellobiohydrolase (reducing end; CBH) | 3.2.1.176 | GH7 | Hydrolytic cleavage/release of cellobiose units from the reducing ends of cellulose chains (exocellulase) |
| β-1,4-endoglucanase | 3.2.1.4 | GH45 | Hydrolytic cleavage of β-1-4 glycosidic bonds within cellulose chains in amorphic regions (endocellulase) |
| ***Hemicelluloses (xylan, glucomannan)*** | | | |
| β-1,4-xylosidase | 3.2.1.37 | GH3 subtypes e0, e21, e73, e88 | Release of D-xylose units by hydrolysis of terminal β-1-4 glycosidic bonds from the non-reducing ends of the xylan backbone and xylo-oligosaccharides (exoxylanase) |
| β-1,4-mannan-cleaving enzymes | 3.2.1.78, 3.2.1.25 | GH5_7 | Hydrolytic degradation or modification of β-mannan-containing polysaccharides (β-mannosidase and endomannosidase activities) |
| β-1,4-endoxylanase | 3.2.1.8 | GH10  GH11 | Hydrolytic cleavage of β-1-4 glycosidic bonds within the xylan backbone under formation of xylo-oligosaccharide fragments (endoxylanase) |
| Xyloglucanase (xyloglucan-specific endo-β-1,4-glucanase) | 3.2.1.151 | GH74 | Endohydrolysis of xyloglucans under formation of xyloglucan fragments (xylogluco-oligosaccharides) |
| Acetyl xylan esterase; feruloyl esterase | 3.1.1.72 3.1.1.73 | CE1 | Hydrolytic deacytelation of xylans and xylo-oligosaccharides (release of acetic acid);  hydrolytic cleavage and release of ferulic acid from the end of hemicellulose branches that form linkages to lignin |
| Acetylesterase | 3.1.1.6 | CE16 | Unspecific hydrolysis of acetyl esters (release of acetic acid) |

*Supplement Table S3. Overview of metaproteomics and amplicon sequencing analyses performed on 127 deadwood samples from 12 tree species in Hainich National Park and the surrounding Dün region. Includes the number of peptides and protein groups identified in the metaproteomics analysis for both fungi and prokaryotes. The number of operational taxonomic units (OTUs) of the corresponding amplicon sequencing analyses from the same samples is also listed.*

| **Metaproteomics** | **Fungi** | **Bacteria** | **Sum** |
| --- | --- | --- | --- |
| **Peptides** | 179,813 | 42,567 | 223,030 |
| **Protein groups** | 97,490 | 11,476 | 109,305 |
| **Amplicon sequencing** | **Fungi** | - | - |
| **OTUs** | 1,184 | - | - |

*Supplement Table S4. Manteltest for the comparison of amplicon sequencing and metaproteomics data matrices (abundance) based on fungal genera. Only fungal genera identified using both methods were used for Spearman correlation. For an overview, see Supplement Fig. S2.*

|  | **Mantel statistic** | |  | **Upper quantiles of permutation** | | | |
| --- | --- | --- | --- | --- | --- | --- | --- |
|  | **Statistic r** | **Significance** |  | **90%** | **95%** | **97.5%** | **99%** |
| **Per Samples** | 0.039 | 0.164 |  | 0.052 | 0.067 | 0.078 | 0.095 |
| **Per Tree Species** | 0.565 | 4×10^-4^ |  | 0.223 | 0.292 | 0.343 | 0.396 |

*Supplement Table S5. PERMANOVA to analyze the impact of tree species, tree clade (angio- vs. gymnosperms), and forest stand (i.e., plot) on the fungal community composition. 127 deadwood samples from 12 tree species of the Hainich National Park and surrounding Dün region (12 plots) were tested by a three-way permutational multivariate analysis of variance (three-way PerMANOVA) based on Bray–Curtis distance for both methods. Amplicon sequencing based on the OTU abundance matrix and metaproteome on the fungal species abundance matrix.*

|  | **Degrees of freedom** | **Sum of square** | ***R*^2^** | ***F* value** | ***p* value** |  |
| --- | --- | --- | --- | --- | --- | --- |
| **Matrix: Samples * Fungi on OTU level** | | | | | | |
| **Tree Species** | 11 | 14.443 | 0.255 | 3.584 | 0.001 | *** |
| **Tree Clade** | 1 | 2.844 | 0.050 | 6.616 | 0.001 | *** |
| **Plot ID** | 11 | 5.063 | 0.089 | 1.027 | 0.332 |  |
| **Matrix: Samples * Lignocellulolytic fungal species (Proteome)** | | | | | | |
| **Tree Species** | 11 | 4.608 | 0.289 | 4.251 | 0.001 | *** |
| **Tree Clade** | 1 | 1.267 | 0.079 | 10.791 | 0.001 | *** |
| **Plot ID** | 11 | 1.907 | 0.12 | 1.421 | 0.016 | * |
| **Matrix: Samples * Lignocellulolytic substrate-specific enzymes (Proteome)** | | | | | | |
| **Tree Species** | 11 | 1.400 | 0.326 | 5.053 | 0.001 | *** |
| **Tree Clade** | 1 | 0.353 | 0.082 | 11.203 | 0.001 | *** |
| **Plot ID** | 11 | 0.211 | 0.049 | 0.54 | 0.98 |  |

Supplement Table S6. Analysis of estimated mass loss for each sample based on a previously published method [35] as part of the BELongDead Experiment. To test the correlation between the estimated mass loss and tree species, tree clade (angio- vs. gymnosperms), forest stand (i.e. plot) or CAZymes (NSAF) and substrate-specific subgroups, an ANOVA based on linear model or linear mixed-effect model (tree species as random factor) was performed for the metaproteomics analysis of 127 deadwood samples from 12 tree species of the Hainich National Park and surrounding Dün region (12 plot).

|  |  | **Degrees of freedom** | **Sum of square** | **Mean of square** | ***F* value** | ***p* value** |  |
| --- | --- | --- | --- | --- | --- | --- | --- |
| **lm(Massloss ~ Tree Species, data)** | | | | | | | |
|  |  | 11 | 1.367 | 0.123 | 22.97 | 2.2x10^-16^ | *** |
| **lm(Massloss ~ Tree Clade, data)** | | | | | | | |
|  |  | 1 | 0.463 | 0.463 | 37.79 | 9.9x10^-9^ | *** |
| **lm(Massloss ~ Plot_ID, data)** | | | | | | | |
|  |  | 11 | 0.083 | 0.008 | 0.454 | 0.927 |  |
| **lm(Massloss ~ Sum CAZymes (NSAF), data)** | | | | | | | |
|  |  | 1 | 0.124 | 0.124 | 8.246 | 0.00481 | ** |
|  | **Sum of square** | **Mean of Square** | **Numerator degrees of freedom** | **Denominator degrees of freedom** | ***F* value** | ***p* value** |  |
| **lmer(Massloss ~ Sum CAZymes (NSAF) + (1\|Tree Species), data)** | | | | | | | |
|  | 0.004 | 0.004 | 1 | 120.0 | 0.748 | 0.3888 |  |
| **lmer(Massloss ~ Lignin (NSAF)** **+ (1\|Tree Species), data)** | | | | | | | |
|  | 6.92x10^-7^ | 6.92x10^-7^ | 1 | 117.0 | 0.0004 | 0.991 |  |
| **lmer(Massloss ~ Lignocellulose (NSAF)** **+ (1\|Tree Species), data)** | | | | | | | |
|  | 0.0032 | 0.0032 | 1 | 118.0 | 0.6000 | 0.4401 |  |
| **lmer(Massloss ~ Cellulose (NSAF)** **+ (1\|Tree Species), data)** | | | | | | | |
|  | 0.0028 | 0.0028 | 1 | 121.0 | 0.5110 | 0.4761 |  |
| **lmer(Massloss ~ Hemicellulose (NSAF)** **+ (1\|Tree Species), data)** | | | | | | | |
|  | 0.0087 | 0.0087 | 1 | 117.3 | 1.6203 | 0.2056 |  |

Supplement Table S7. Correlation between species richness and tree species, tree clade (angio- vs. gymnosperms), forest stand (i.e., plot). ANOVA based on a linear model for the observed species richness based on OTUs of amplicon sequencing or fungal species of metaproteomics of 127 deadwood samples from the 12 tree species of the Hainich National Park and surrounding Dün region (12 plot). Prior to ANOVA, the species richness of the metaproteomics was transformed to correct its left-skewed distribution.

|  |  | **Degrees of freedom** | **Sum of square** | **Mean of square** | ***F* value** | ***p* value** |  |
| --- | --- | --- | --- | --- | --- | --- | --- |
| **Amplicon sequencing** | | | | | | | |
| **lm(Species Richness ~ Tree Species, data)** | | | | | | | |
|  |  | 11 | 10412 | 946.6 | 4.194 | 3.3x10^-5^ | *** |
| **lm(Species Richness ~ Tree Clade, data)** | | | | | | | |
|  |  | 1 | 4027 | 4027 | 15.55 | 0.0001 | *** |
| **lm(Species Richness~ Plot ID, data)** | | | | | | | |
|  |  | 11 | 5167 | 469.8 | 1.729 | 0.0756 |  |
| **Metaproteome** | | | | | | | |
| **lm(Species Richness ~ Tree Species, data)** | | | | | | | |
|  |  | 11 | 3.588 | 0.326 | 2.702 | 0.004 | ** |
| **lm(Species Richness ~ Tree Clade, data)** | | | | | | | |
|  |  | 1 | 0.039 | 0.039 | 0.281 | 0.597 |  |
| **lm(Species Richness ~ Plot ID, data)** | | | | | | | |
|  |  | 11 | 1.626 | 0.147 | 1.071 | 0.391 |  |

Supplement Table S8. Effect of fungal amplicon sequencing- and metaproteomics-based species richness on the CAZymes (NSAF) and substrate-specific subgroups divided into lignin and/or other aromatics, cellulose, and hemicelluloses, or exhibit supportive activities related to lignocellulose modification and degradation (lignocellulose-associated, H_2_O_2_-forming). Species richness was calculated for amplicon sequencing per OTUs and metaproteomics per fungal species. Results are based on a linear mixed-effect model with tree species as a random factor and the corresponding ANOVA of 127 deadwood samples from 12 tree species of the Hainich National Park and surrounding Dün region (12 plots).

|  | **Sum of square** | **Numerator degrees of freedom** | **Denominator degrees of freedom** | ***F* value** | ***p* value** | **Estimates** |
| --- | --- | --- | --- | --- | --- | --- |
| **Sum CAZymes.** lmer(species richness ~ Sum CAZymes + (1\|Tree Species), data) | | | | | | |
| Species Richness (OTU) | 89193 | 1 | 125.26 | 3.59 | 0.060 | - |
| Species Richness (Proteome) | 512284 | 1 | 121.33 | 24.39 | 2.54x10^-6^ | + |
|  |  |  |  |  |  |  |
| **Lignin:** lmer(species richness ~ Lignin + (1\|Tree Species), data) | | | | | | |
| Species Richness (OTU) | 15301 | 1 | 125.38 | 5.33 | 0.023 | - |
| Species Richness (Proteome) | 13637 | 1 | 124.74 | 4.77 | 0.031 | + |
|  |  |  |  |  |  |  |
| **Lignocellulose:** lmer(species richness ~ Lignocellulose + (1\|Tree Species), data) | | | | | | |
| Species Richness (OTU) | 1209 | 1 | 125.86 | 0.43 | 0.513 | - |
| Species Richness (Proteome) | 143250 | 1 | 122.14 | 90.66 | 2.2x10^-16^ | + |
|  |  |  |  |  |  |  |
| **Cellulose:** lmer(species richness ~ Cellulose + (1\|Tree Species), data) | | | | | | |
| Species Richness (OTU) | 10410 | 1 | 123.68 | 1.20 | 0.275 | - |
| Species Richness (Proteome) | 32221 | 1 | 120.53 | 3.81 | 0.053 | + |
|  |  |  |  |  |  |  |
| **Hemicellulose:** lmer(species richness ~ Hemicellulose + (1\|Tree Species), data) | | | | | | |
| Species Richness (OTU) | 2345 | 1 | 124.13 | 4.78 | 0.031 | - |
| Species Richness (Proteome) | 1444 | 1 | 125.44 | 2.93 | 0.090 | + |
|  |  |  |  |  |  |  |

Supplement Table S9. ANOVA is based on a linear model to test the effect of tree clade and tree species on community-level functional redundancy, according to Ricotta and Pavoine [55]. Functional redundancy was calculated for all CAZymes (NSAF) as well as separately for the substrate-specific subgroups, i.e., lignin and/or other aromatics, cellulose, and hemicelluloses, or supporting activities related to lignocellulose modification and degradation (lignocellulose-associated, H_2_O_2_-forming).

|  | **Degrees of freedom** | **Sum of square** | **Mean of square** | ***F* value** | ***p* value** |  |
| --- | --- | --- | --- | --- | --- | --- |
| **Sum CAZyme** | | | | | | |
| **lm(Functional Redundancy ~ Tree Clade, data)** | | | | | | |
|  | 1 | 0.016 | 0.016 | 10.86 | 0.0013 | ** |
| **lm(Functional Redundancy ~ Tree Species, data)** | | | | | | |
|  | 11 | 0.058 | 0.005 | 4.37 | 1.8x10^-5^ | *** |
|  | **Significant/marginal significant Coefficients** | | | |  |  |
|  | *Fraxinus* |  |  |  | 0.0369 | * |
|  | *Picea* |  |  |  | 0.0175 | * |
|  | *Fagus* |  |  |  | 0.0638 | . |
|  | *Pseudotsuga* |  |  |  | 0.0971 | . |
|  | *Tilia* |  |  |  | 0.0829 | . |
|  |  |  |  |  |  |  |
|  | **Sum of square** | **Numerator degrees of freedom** | **Denominator degrees of freedom** | ***F* value** | ***p* value** |  |
| **Lignin**  **lm(Functional Redundancy ~ Tree Clade, data)** | | | | | | |
|  | 1 | 0.024 | 0.024 | 2.662 | 0.105 |  |
| **lm(Functional Redundancy ~ Tree Species, data)** | | | | | | |
|  | 11 | 0.157 | 0.014 | 1.657 | 0.0927 |  |
| **Lignocellulose**  **lm(Functional Redundancy ~ Tree Clade, data)** | | | | | | |
|  | 1 | 0.05 | 0.05 | 9.477 | 0.0026 | ** |
| **lm(Functional Redundancy ~ Tree Species, data)** | | | | | | |
|  | 11 | 0.2 | 0.018 | 4.024 | 5.6x10^-5^ | *** |
| **Cellulose**  **lm(Functional Redundancy ~ Tree Clade, data)** | | | | | | |
|  | 1 | 0.012 | 0.012 | 8.98 | 0.0033 | ** |
| **lm(Functional Redundancy ~ Tree Species, data)** | | | | | | |
|  | 11 | 0.041 | 0.004 | 3.131 | 0.0010 | *** |
| **Hemicellulose**  **lm(Functional Redundancy ~ Tree Clade, data)** | | | | | | |
|  | 1 | 0.324 | 0.324 | 20.76 | 1.2x10^-5^ | *** |
| **lm(Functional Redundancy ~ Tree Species, data)** | | | | | |  |
|  | 11 | 0.499 | 0.0453 | 2.93 | 0.0019 | ** |

1. **Supplementary figures**


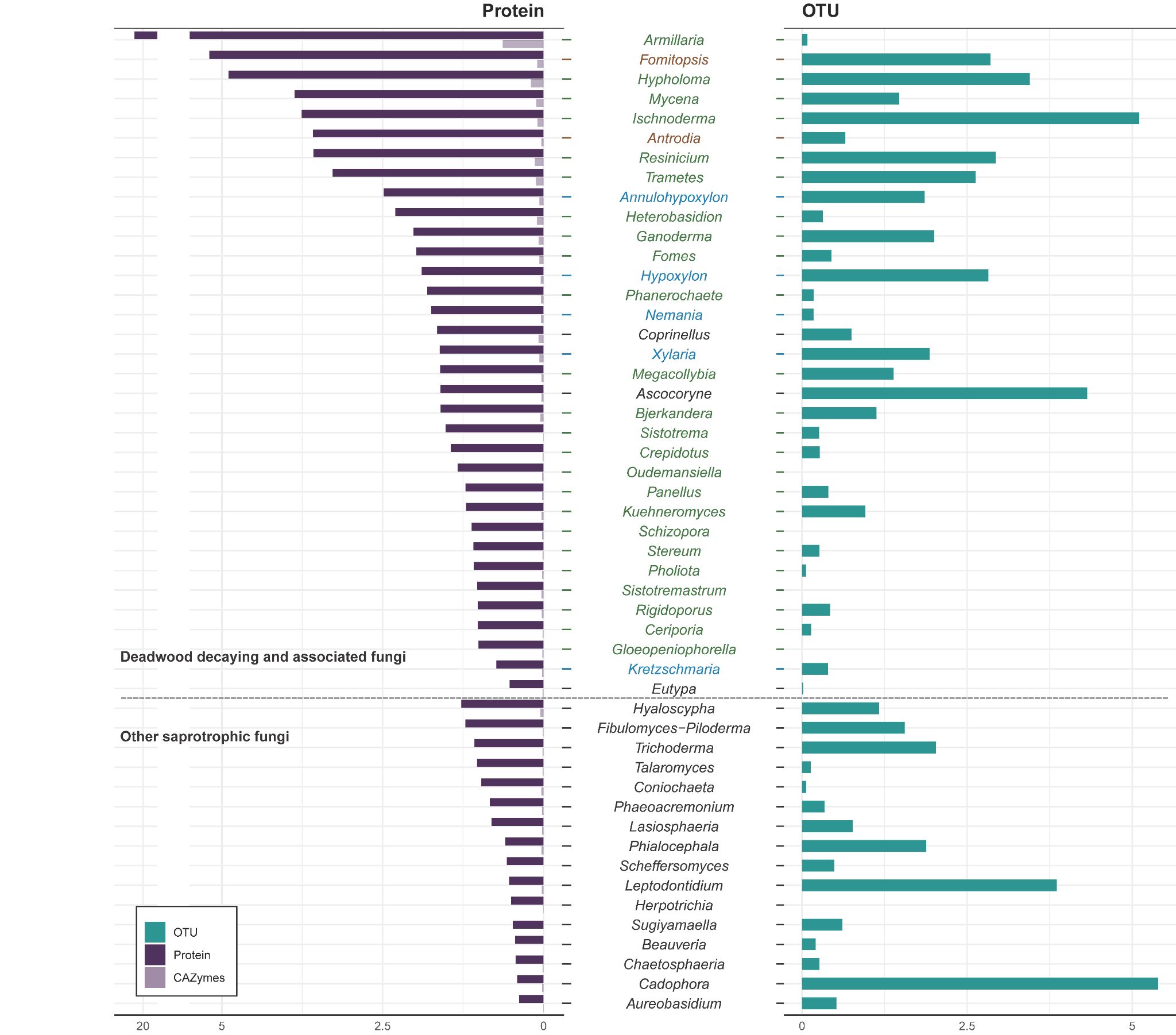


*Supplement Figure S1. Comparison of 50 fungal genera based on their relative abundances in OTUs (Operational Taxonomic Units) determined by amplicon sequencing (green bars) and their corresponding relative protein abundances in metaproteomics (light purple bars). In addition, the relative abundances of fungi producing lignocellulolytic CAZymes are shown in light purple. White-rot fungi (WRF) are shown in green, brown-rot fungi (BRF) in brown, soft-rot fungi (SRF) in blue, and other saprotrophic fungal genera in black.*


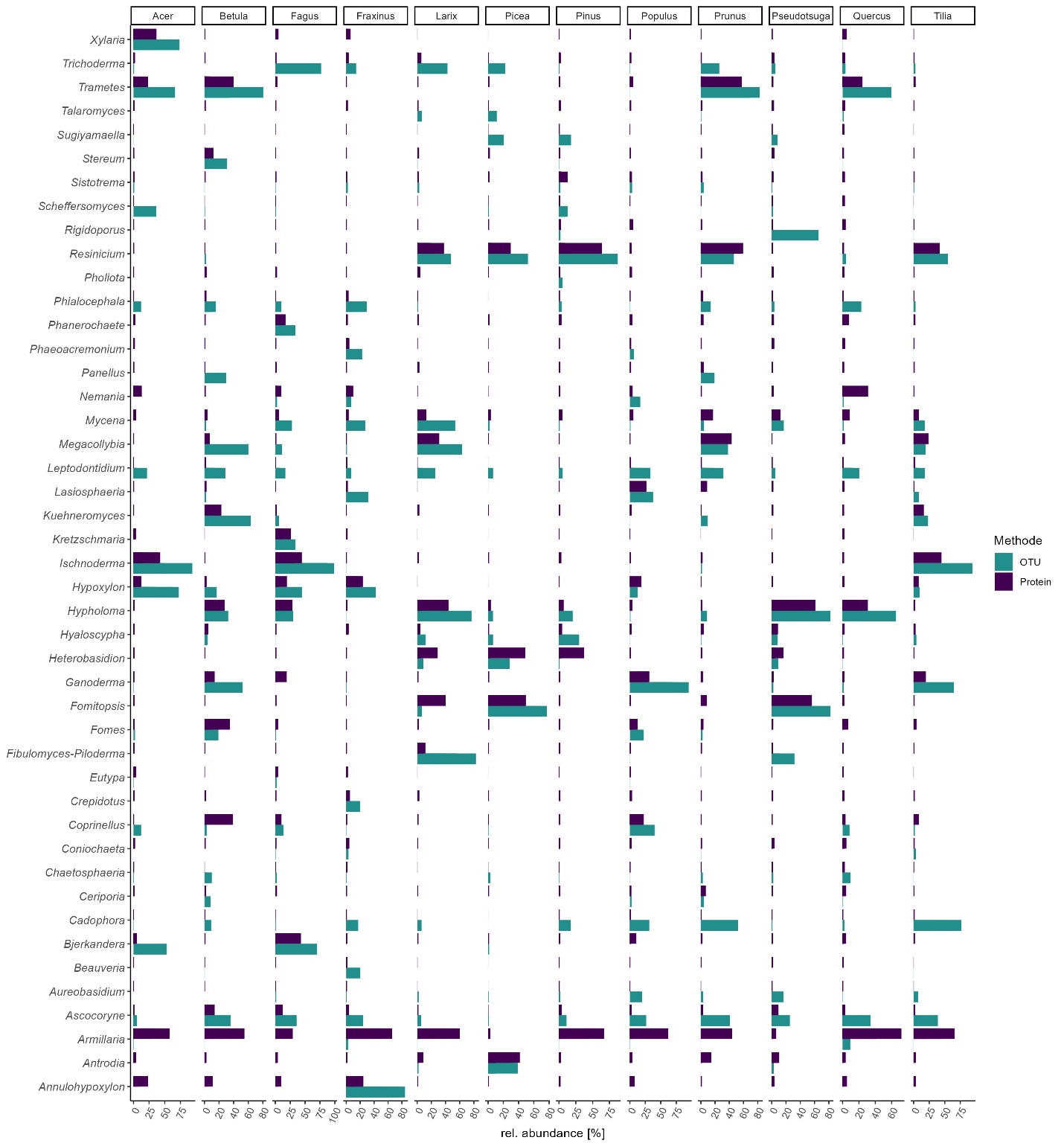
 *Supplement Figure S2. Comparison of relative abundance of selected fungal genera unique between the amplicon sequencing and metaproteomics approach. The mean relative abundance of OTU (operational taxonomic unit) or NSAF (normalized spectral abundance factor) is shown per deadwood tree species.*


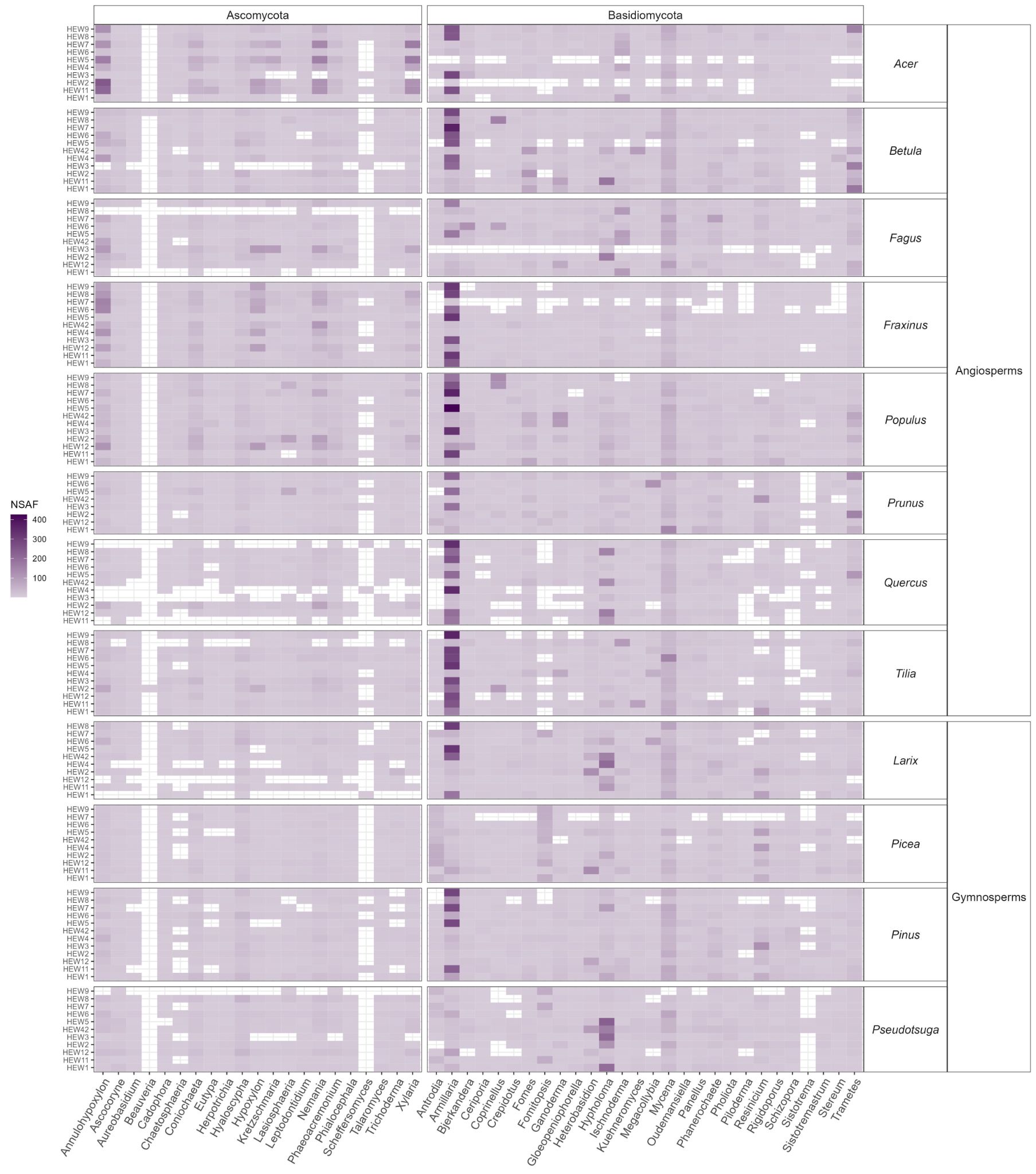
 *Supplement Figure S3. Heatmap showing the normalized spectral abundance factor (NSAF) of fungal genera producing lignocellulolytic CAZymes. The data include 127 deadwood samples taken from 12 tree species and 12 different plots in Hainich National Park and the surrounding Dün exploratory.*


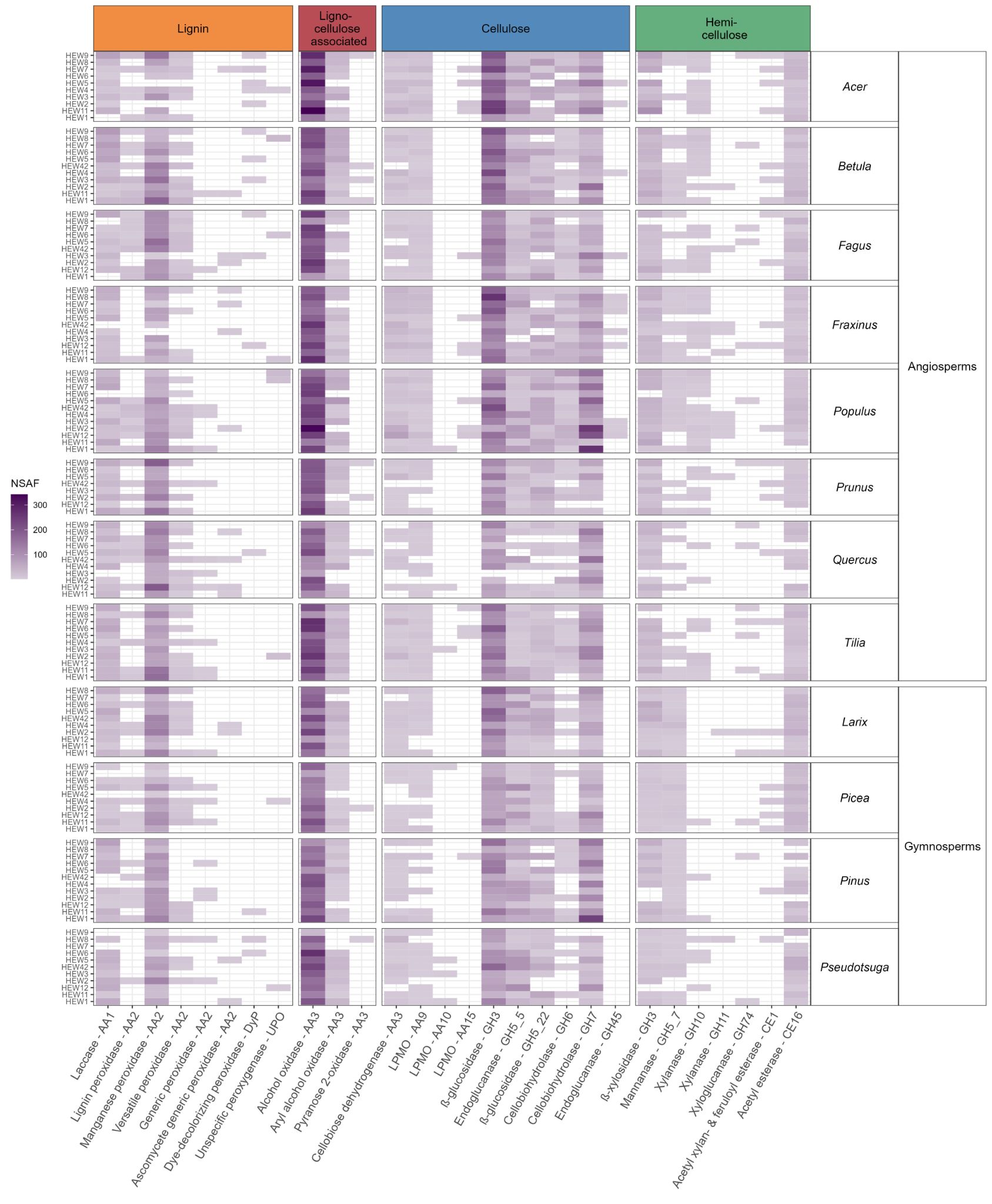
 *Supplement Figure S4. Heatmap showing the normalized spectral abundance factor (NSAF) of selected fungal CAZymes that act on lignin and/or other aromatics, cellulose, and hemicelluloses, or exhibit supportive activities related to lignocellulose modification and degradation (lignocellulose-associated, H_2_O_2_-forming), calculated for the analyzed 127 samples in the Hainich National Park and surrounding Dün exploratory.*


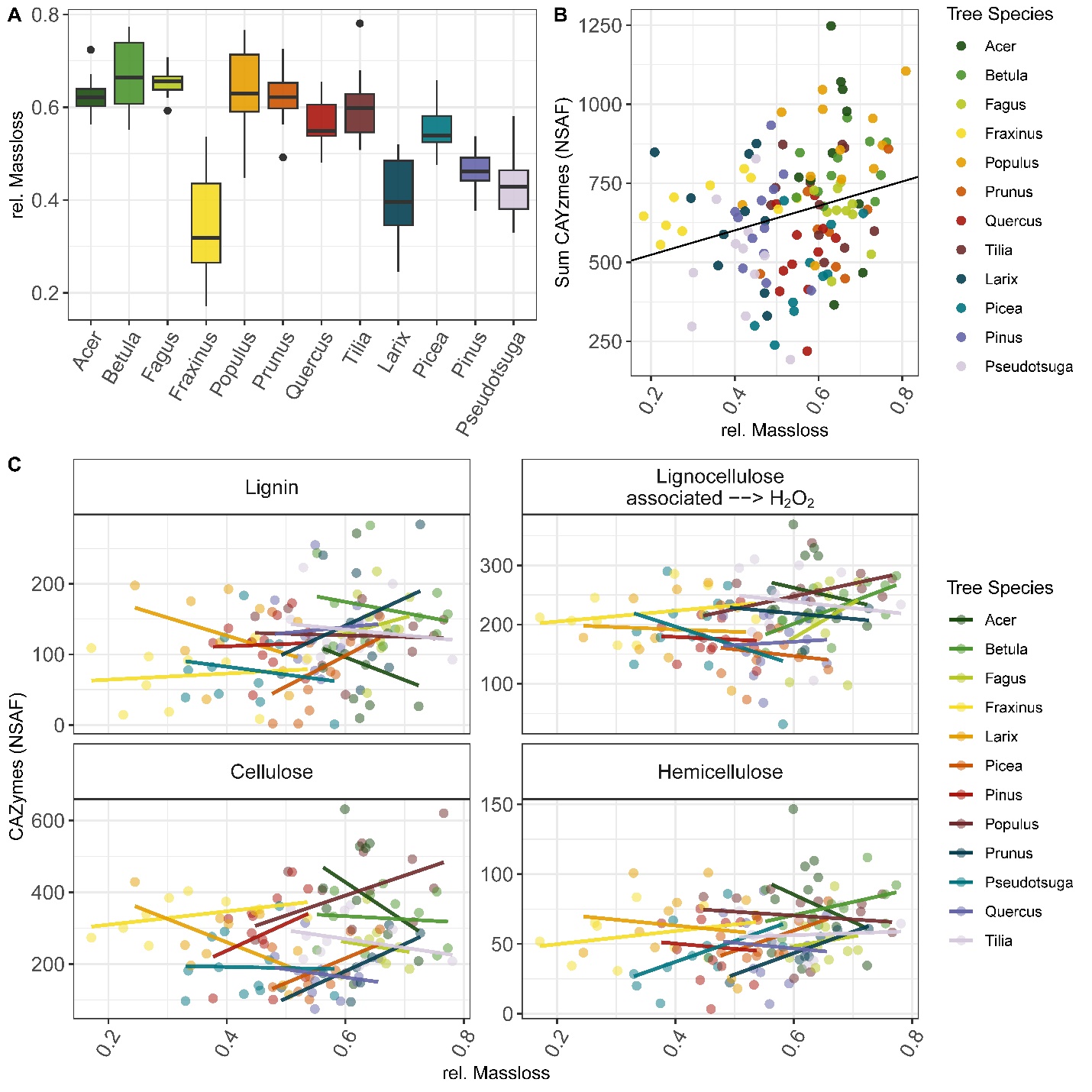


Supplement Figure S5. Analysis of estimated mass loss for each sample based on a previously published method [35] as part of the BELongDead Experiment. Boxplots show the relative mass loss per tree species (A). Relationship between relative mass loss and CAZymes (B) or for substrate-specific subgroups, including lignin and/or other aromatics, cellulose and hemicelluloses, or exhibit supportive activities related to lignocellulose modification and degradation (lignocellulose-associated, H_2_O_2_-forming) were performed over samples (C), separated by color for the tree species. Regression line (B) represents the slope of a linear model that shows a significant interaction (p = 0.0048) between mass loss and CAZymes, independent of tree species. Relationship of mass loss and separate per substrate-specific enzyme class (C) shows regression lines per tree species. An associated linear mixed-effect model with random factor tree species does not reveal significant interaction. For details of statistical analysis, see **Supplement Tab. S6**.


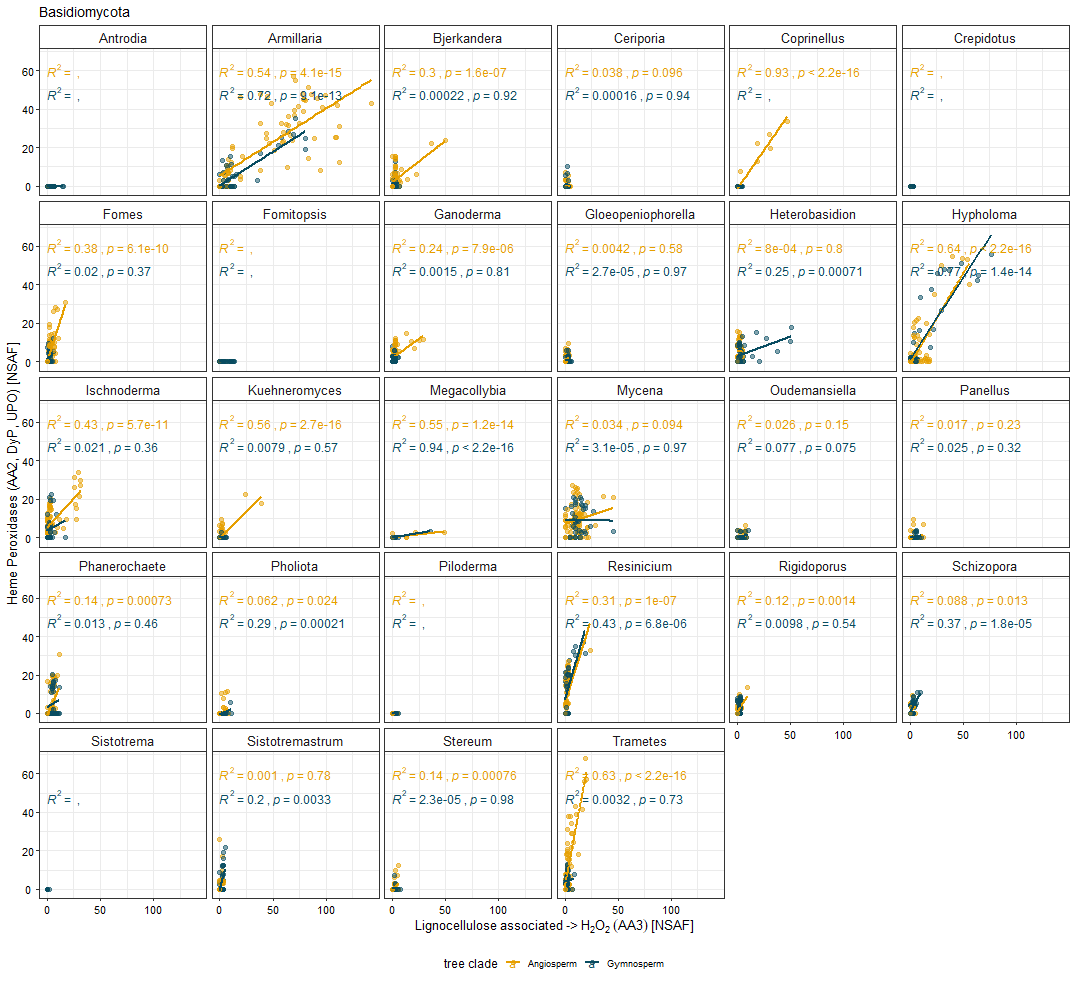


Supplement Figure S6. Correlations of lignin-related activity (peroxide-producing enzymes vs. peroxidases) of basidiomycetous genera. The data points represent the cumulative normalized spectral abundance factor (NSAF) of the different enzymes per sample, separated by fungal genus and colored within the plots according to occurrence in angiosperm (yellow) and gymnosperm logs (blue).


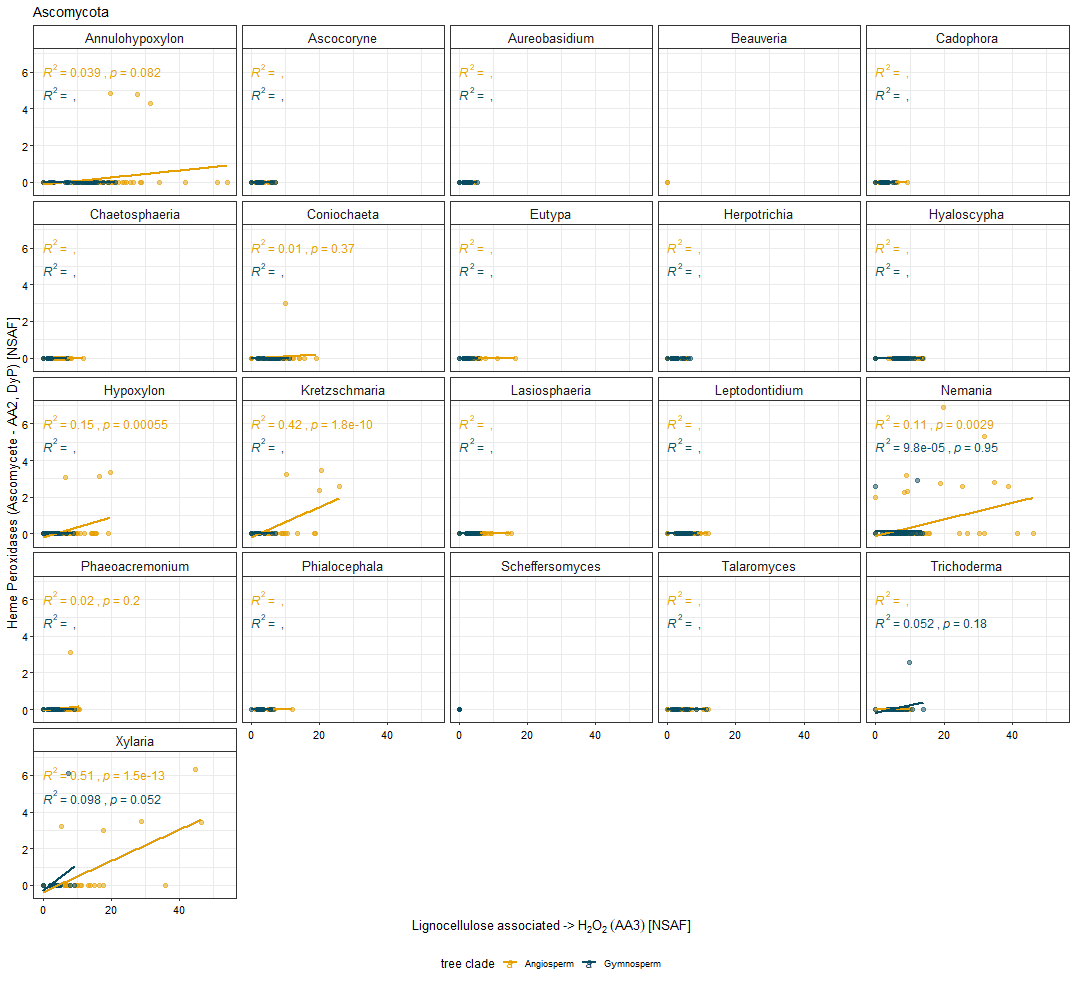


Supplement Figure S7. Correlations of lignin-related activity (peroxide-producing enzymes vs. peroxidases) of ascomycetous genera. The data points represent the cumulative normalized spectral abundance factor (NSAF) of the different enzymes per sample, separated by fungal genus and colored within the plots according to occurrence in angiosperm (yellow) and gymnosperm logs (blue).


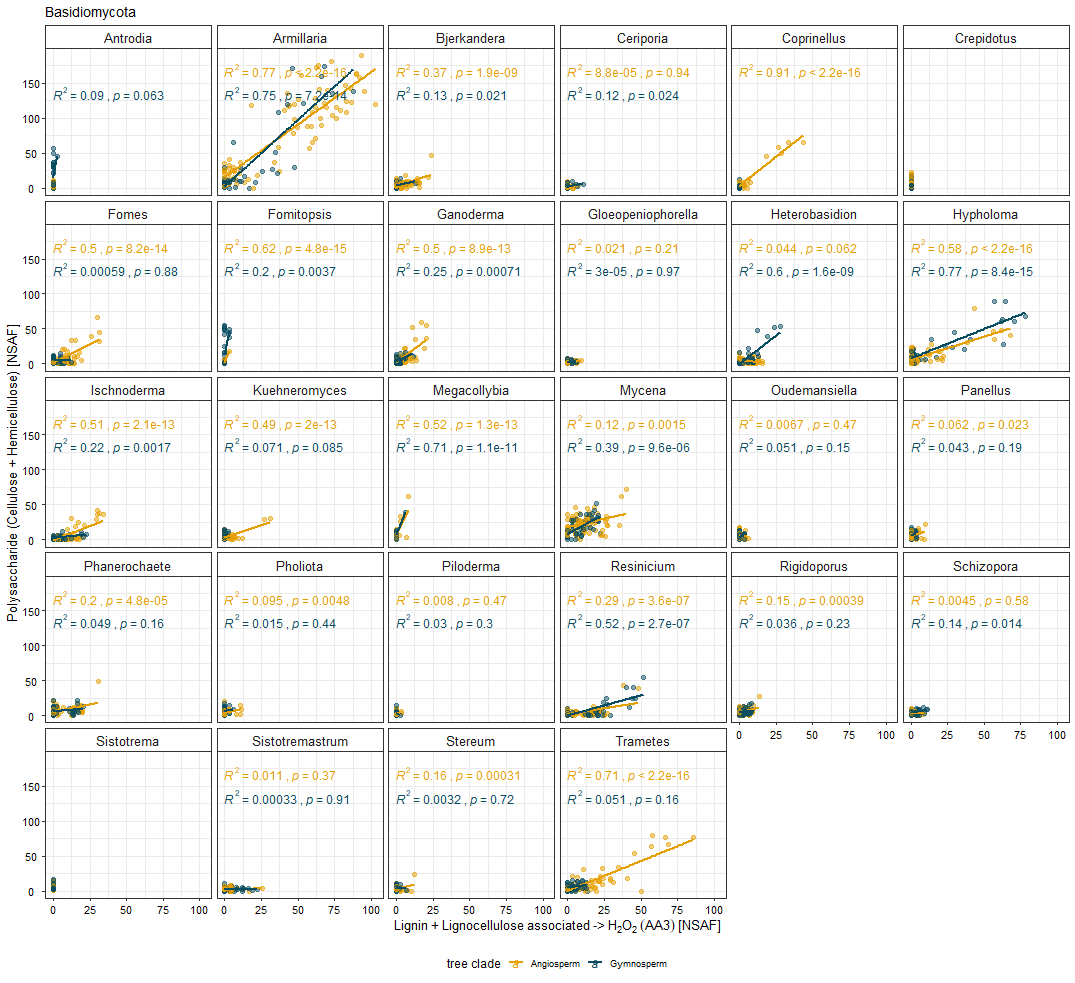


Supplement Figure S8. Correlations of enzymes modifying lignin and polysaccharides (cellulose, hemicelluloses) of basidiomycetous genera. The data points represent the cumulative normalized spectral abundance factor (NSAF) of the different enzymes per sample, separated by fungal genus and colored within the plots according to occurrence in angiosperm (yellow) and gymnosperm logs (blue).


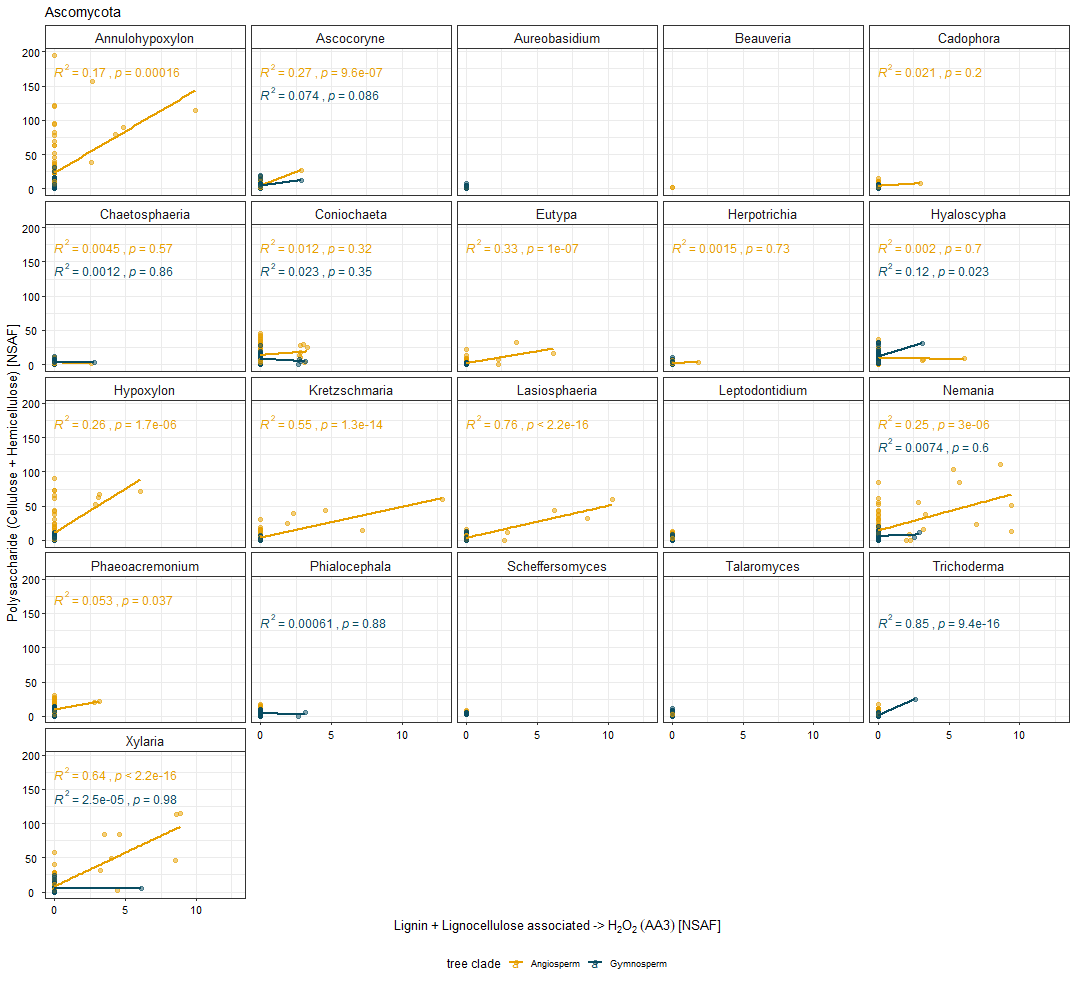


Supplement Figure S9. Correlations of enzymes modifying lignin and polysaccharides (cellulose, hemicelluloses) of ascomycetous genera. The data points represent the cumulative normalized spectral abundance factor (NSAF) of the different enzymes per sample, separated by fungal genus and colored within the plots according to occurrence in angiosperm (yellow) and gymnosperm logs (blue).


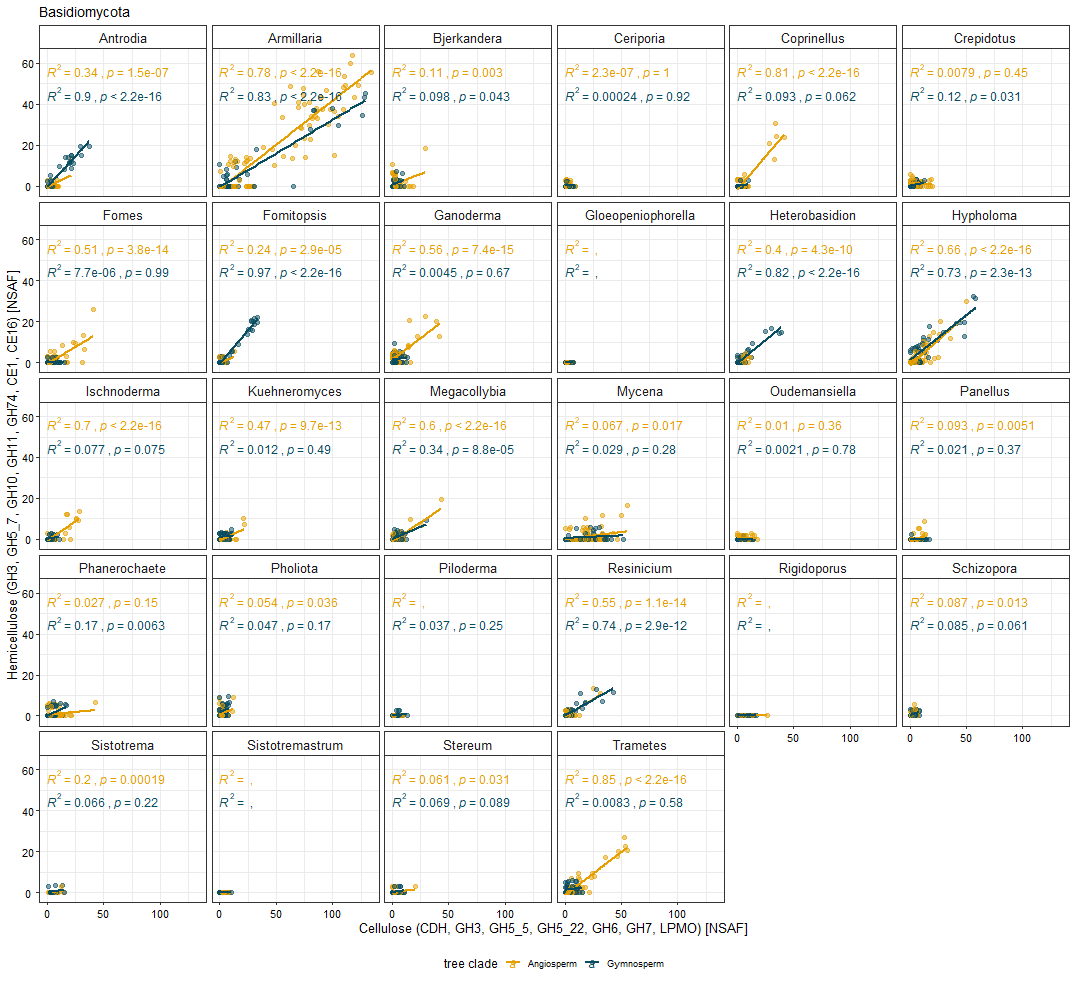


Supplement Figure S10. Correlations of cellulolytic and hemicellulolytic enzymes of basidiomycetous genera. The data points represent the cumulative normalized spectral abundance factor (NSAF) of the different enzymes per sample, separated by fungal genus and colored within the plots according to occurrence in angiosperm (yellow) and gymnosperm logs (blue).


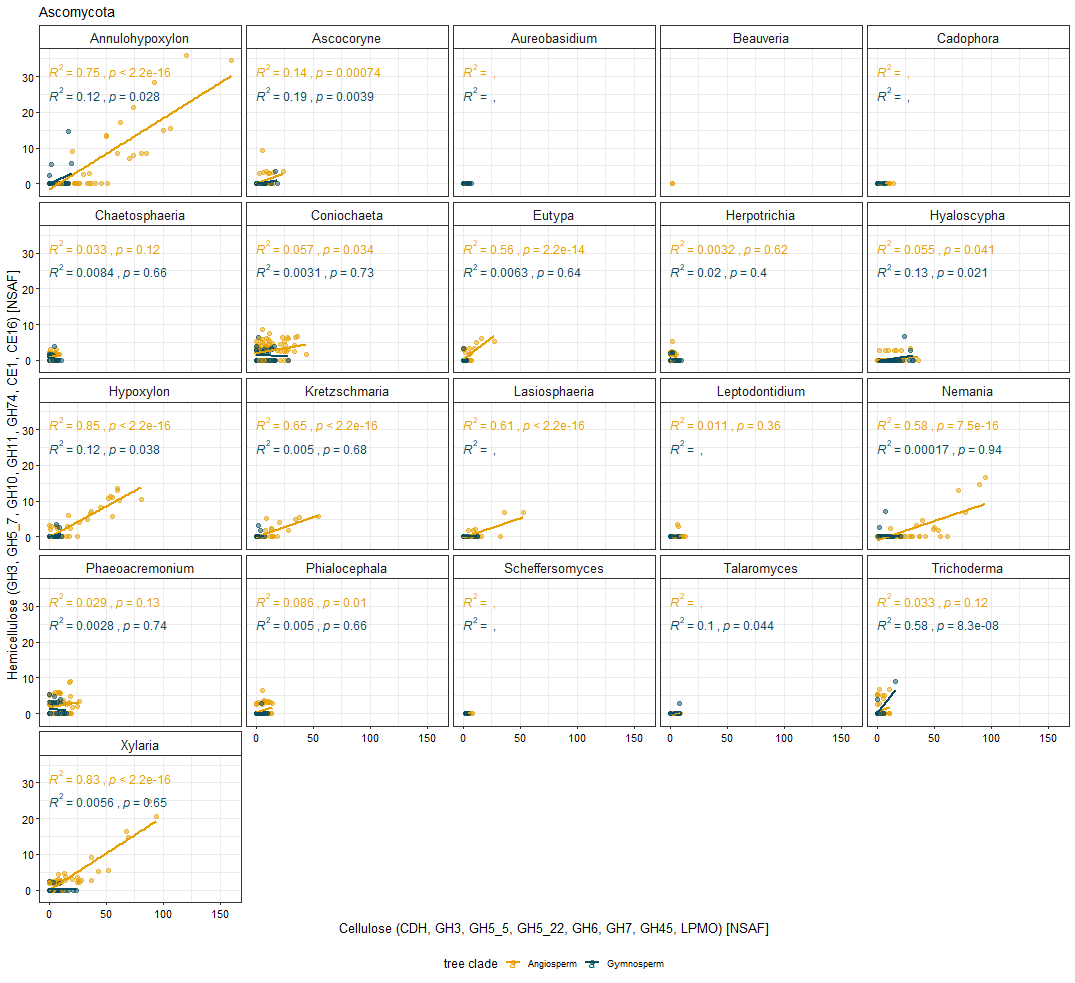


Supplement Figure S11. Correlations of cellulolytic and hemicellulolytic enzymes of ascomycetous genera. The data points represent the cumulative normalized spectral abundance factor (NSAF) of the different enzymes per sample, separated by fungal genus and colored within the plots according to occurrence in angiosperm (yellow) and gymnosperm logs (blue).


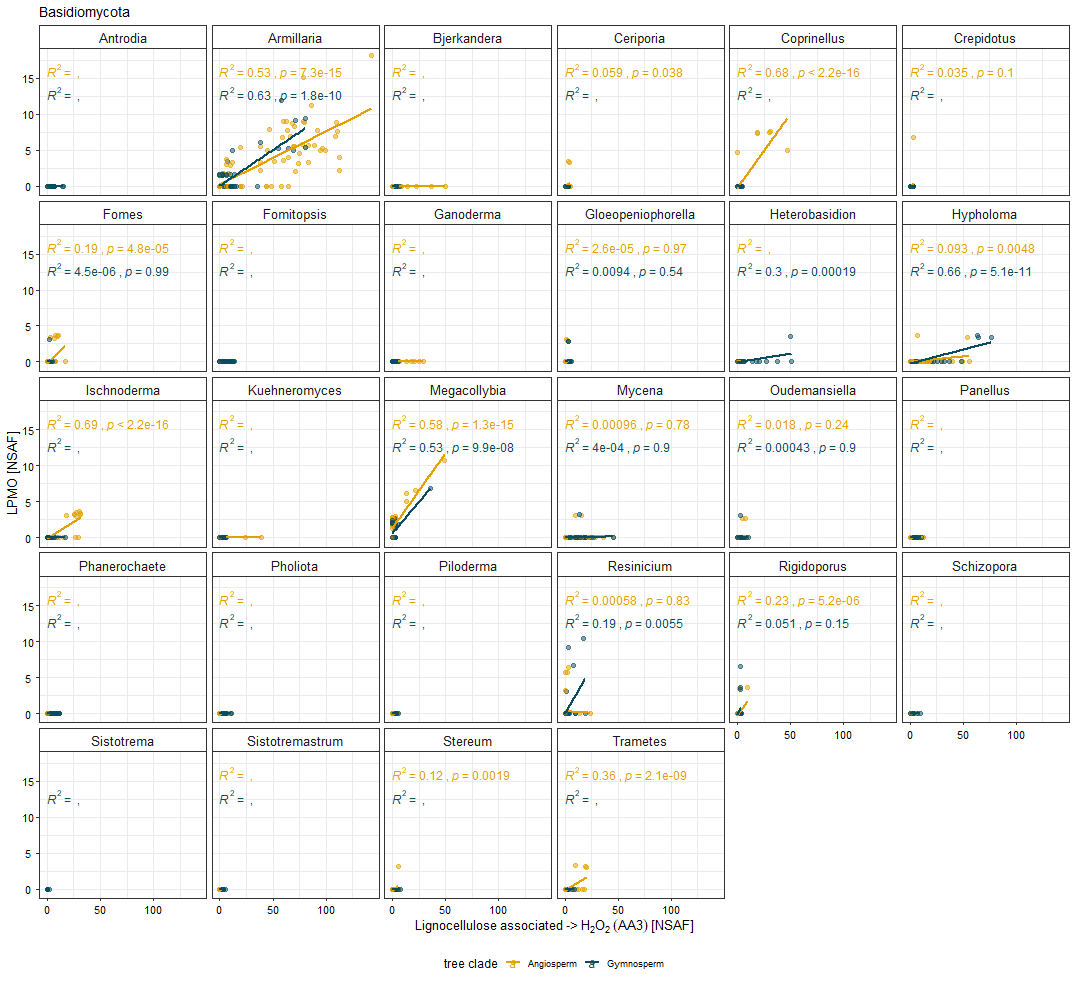


Supplement Figure S12. Correlations of peroxide-producing (AA3) enzymes and LPMOs of basidiomycetous genera. The data points represent the cumulative normalized spectral abundance factor (NSAF) of the different enzymes per sample, separated by fungal genus and colored within the plots according to occurrence in angiosperm (yellow) and gymnosperm logs (blue).


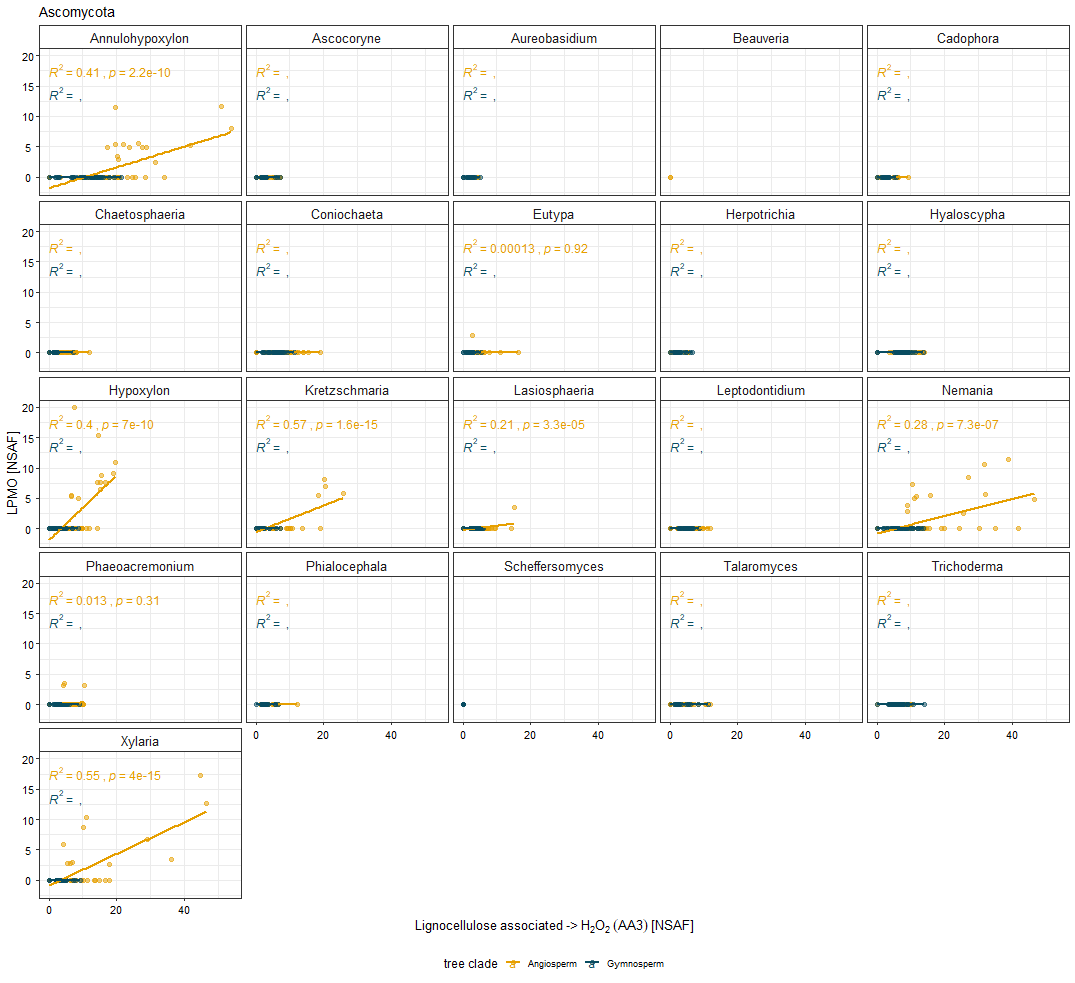


Supplement Figure S13. Correlations of peroxide-producing (AA3) enzymes and LPMOs of ascomycetous genera. The data points represent the cumulative normalized spectral abundance factor (NSAF) of the different enzymes per sample, separated by fungal genus and colored within the plots according to occurrence in angiosperm (yellow) and gymnosperm logs (blue).

**Supplementary references**

95. Sievers F, Higgins DG. Clustal Omega for making accurate alignments of many protein sequences. *Protein Sci*. 2018; 27:135–145.

96. Aspeborg H, Coutinho PM, Wang Y, Brumer H, Henrissat B. Evolution, substrate specificity and subfamily classification of glycoside hydrolase family 5 (GH5). *BMC Evol Biol.* 2012; 12:186.
